# Molecular analysis of the massive GSH transport mechanism mediated by the human Multidrug Resistant Protein 1/ABCC1

**DOI:** 10.1038/s41598-020-64400-x

**Published:** 2020-05-06

**Authors:** Rachad Nasr, Doriane Lorendeau, Ruttiros Khonkarn, Lauriane Dury, Basile Pérès, Ahcène Boumendjel, Jean-Claude Cortay, Pierre Falson, Vincent Chaptal, Hélène Baubichon-Cortay

**Affiliations:** 1 Drug Resistance and Membrane Proteins group, IBCP, UMR 5086, CNRS-University of Lyon, 69367 Lyon, France; 2 Department of Molecular Pharmacochemistry (DPM), UMR 5063, Grenoble Alpes University, 38041 Grenoble, France; 3 INSERM U1052, CNRS-University of Lyon UMR-5286, Cancer Research Center of Lyon (CRCL), 69008 Lyon, France; 4 Present Address: Department of Pharmaceutical Sciences, Faculty of Pharmacy, Chiang Mai University, Chiang Mai, 50200 Thailand

**Keywords:** Biochemistry, Biological techniques, Molecular biology

## Abstract

The transporter Multidrug Resistance Protein 1 (MRP1, ABCC1) is implicated in multidrug resistant (MDR) phenotype of cancer cells. Glutathione (GSH) plays a key role in MRP1 transport activities. In addition, a ligand-stimulated GSH transport which triggers the death of cells overexpressing MRP1, by collateral sensitivity (CS), has been described. This CS could be a way to overcome the poor prognosis for patients suffering from a chemoresistant cancer. The molecular mechanism of such massive GSH transport and its connection to the other transport activities of MRP1 are unknown. In this context, we generated MRP1/MRP2 chimeras covering different regions, MRP2 being a close homolog that does not trigger CS. The one encompassing helices 16 and 17 led to the loss of CS and MDR phenotype without altering basal GSH transport. Within this region, the sole restoration of the original G1228 (D1236 in MRP2) close to the extracellular loop between the two helices fully rescued the CS (massive GSH efflux and cell death) but not the MDR phenotype. The flexibility of that loop and the binding of a CS agent like verapamil could favor a particular conformation for the massive transport of GSH, not related to other transport activities of MRP1.

## Introduction

Cancer cells develop resistance to chemotherapeutic treatments through different mechanisms. A concomitant overexpression of ATP-binding Cassette (ABC) transporters is often observed. Three ABC transporters are most implicated in this phenotype, ABCB1 or P-gp (P-glycoprotein), ABCG2 or BCRP (Breast Cancer Resistant Protein) and ABCC1 or MRP1 (Multidrug Resistance Protein 1). The structural unit of these transporters is composed of a transmembrane domain (TMD) that binds substrates and a nucleotide binding domain (NBD) for ATP hydrolysis. As these pumps translocate structurally-unrelated drugs, their overexpression triggered by exposure of cancer cells to one drug leads to the resistance to several others, thereby conferring a multidrug resistance (MDR) phenotype. MRP1 is involved in inflammation, detoxification and oxidative stress^1,2^. A high level of MRP1 expression was associated with poor clinical outcome of children with neuroblastoma^3^. Glutathione (GSH) plays a key role in the transport activity carried out by MRP1. Indeed, MRP1 transports GS-conjugated anions like Leukotriene C_4_ (LTC_4_), a physiological substrate transported with a high affinity^4^. Moreover, MRP1 co-transports anticancer drugs such as doxorubicin, vincristine, and etoposide with GSH^5,6^. Finally, MRP1 pumps out reduced GSH with a low affinity and its oxidized form, GSSG, with a higher affinity^7^. GSH is a tripeptide that plays a key role in protecting cells against oxidative stress making it a vital compound in the cell. Importantly, the transport of GSH by MRP1 can be greatly stimulated by modulators such as verapamil^8^. Furthermore, GSH itself stimulates estrone sulfate transport^9^. This latter activity has been mechanically distinguished from the basal activity of GSH transport^10^. Yet, the structural determinants that differentiate the two modes of transport of GSH by MRP1, and in particular the regions specific of the stimulated mode, remain largely unknown.

We have shown that the stimulation of GSH efflux by verapamil, a MRP1 ligand^11^, triggers the selective cell death of BHK21 (Baby Hamster Kidney 21) cells overexpressing MRP1 following a rapid and massive depletion of cellular GSH^12^. This led us to propose and develop a strategy that was first discovered in 1952 by Szybalski and Bryson^13^; it is called “Collateral Sensitivity (CS)”^14^. The overexpression of MRP1 representing the strength of cancer cells can be exploited as a weakness by some CS agents, such as verapamil, that specifically target this transporter. In this way, MRP1 becomes the “Achilles’ heel” of these resistant cells by triggering a lethal oxidative stress^14,15^. MRP1, when overexpressed, was shown to regulate basal and apoptotic GSH release, suggesting that it plays a key role in these processes^16^. Recently, it was also identified as an actor of ferroptosis by regulating intracellular glutathione abundance^17^.

Beside verapamil, which is known to exhibit cardiotoxic properties, we identified new CS-triggering agents such as the flavonoid dimer 4e, 50 times more efficient than verapamil^18^.

Identifying the structural determinants of the ligand-stimulated transport of GSH by MRP1 is critical to understand the mechanism by which MRP1 regulates the GSH homeostasis and to allow the design of future CS agents that can selectively kill MRP1-overexpressing chemoresistant cancer cells. To successfully discriminate the regions in MRP1 that selectively control the stimulated mode of GSH transport from the basal transport of GSH, we decided to follow the strategy of chimera constructs to localize and characterize the substrates-binding sites^19,20^. We generated MRP1/MRP2 chimeras, based on their common ability to export GSH, yet with different affinities (*K*_*M*_ of ~20 mM for MRP2 and of 1-5 mM for MRP1^21,22^,) and modulation specificities^23–26^. MRP1 and MRP2 (ABCC2) share 48% of sequence identity and 78% homology and present some similarities in substrate specificity^27^. However, MRP2-mediated GSH transport is poorly stimulated by MRP2 activators and with a spectrum of activators that is different from MRP1^23–26^. Moreover, in polarized cells, although MRP2 is also able of anti-cancer drugs transport, its specificity and affinities are generally different from MRP1^28–30^. Taken together, this suggests that the structural determinants of substrate transport, notably GSH and drugs are different in MRP1 and MRP2. We therefore used a strategy based on MRP1/MRP2 chimeras to screen for regions and residues of MRP1 that are essential for the CS agents-mediated stimulation of GSH efflux and attempted to discriminate these regions from that involved in drug transport. We measured basal and stimulated GSH efflux and drug transport on cells overexpressing MRP1, chimera and mutant proteins. We found a glycine residue near the extracellular loop, solely implicated in the phenomenon of GSH efflux stimulation and collateral sensitivity, discriminating this activity from the others catalyzed by MPR1. In the light of these results, we proposed a mechanistic hypothesis to explain the strong efflux of glutathione observed in the presence of our CS ligands.

## Results

### TM16-TM17 of MRP1 are essential for the GSH-dependent transport of drugs but not for the basal transport of GSH

We undertook to dissect the particular mechanism of massive GSH efflux by studying the implication of the different parts of the transporter MRP1 in this phenomenon and to discriminate the regions in MRP1 that selectively control the stimulated mode of transport of GSH from the basal transport of GSH by using MRP1/MRP2 chimeras.

**Figure 1:**
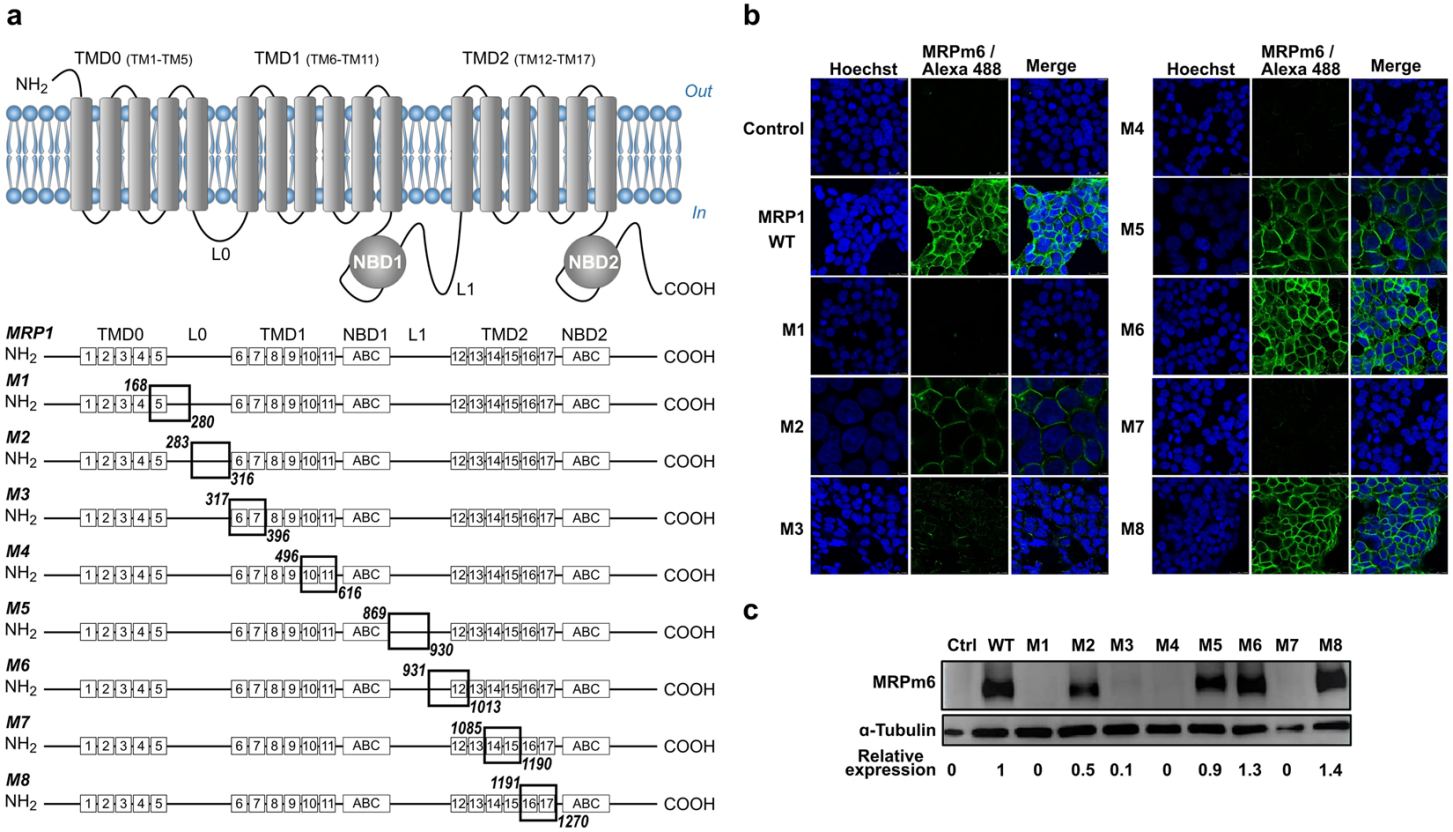
Topology of MRP1 and resulting chimeras expressed in FlpIn 293 cell line. (**a**) Regions of MRP1 exchanged with their MRP2 equivalents in the 8 chimeras. (**b**) Fluorescence microscopy using the MRPm6 antibody and its Alexa 488-conjugated secondary antibody (green). Nuclei are stained with Hoechst 33258 (blue). (**c**) Western blot revealed with MRPm6. The relative level of expression in respect of α-tubulin and the native MRP1 is indicated. The nitrocellulose membrane was cut after the marker 95 kDa and the two parts were separately probed with either the anti-MRP1 monoclonal antibody MRPm6, or a polyclonal alpha-tubulin antibody as loading control. The two parts were re-assembled after cutting and probing. Full-length blot is presented in Supplementary Information.

The borders of regions in MRP1 exchanged with those of MRP2 were defined by sequence alignment and based on the regions pointed out in previous photolabeling studies as essential for the binding of GSH and of the GS-moiety of LTC_4_^31–33^. These regions encompass TM5 (TransMembrane helix 5), L0 (or ICL3 (Intracellular Loop 3)), TM6-TM7, ECL4 (Extracellular Loop 4), TM10-TM11, L1 (or ICL6), TM12-ECL7, and TM16-TM17. The regions we exchanged are summarized in Fig. 1a and detailed in Table 1. They also included the coupling helices ICL5 and ICL7 and their associated TMs 10-11 and 14-15, respectively, due to their role in substrate transport^34,35^. Eight different chimeras were engineered (Fig. 1a and Table 1): M1 (TM5 and the N-terminus half of L0), M2 (the C-terminus of L0), M3 (TM6-TM7), M4 (ICL5 and TM10-TM11), M5 (N-terminus half of L1), M6 (the C-terminus of L1 and TM12), M7 (TM14-TM15 and ICL7) and M8 (TM16-TM17).

**Table 1:** Exchanged fragments in MRP1 with the corresponding MRP2 fragments in MRP1/MRP2 chimeras.

Chimera name	Domain	Exchanged amino-acid Fragments
M1	TM5-L0	MRP1 ^168^D-^280^K → MRP2 ^162^N-^267^Q
M2	L0	MRP1 ^283^A-^316^V → MRP2 ^270^G-^303^A
M3	TM6-TM7	MRP1 ^317^L-^396^K → MRP2 ^304^L-^383^R
M4	ICL5-TM10-TM11	MRP1 ^493^A-^616^L → MRP2 ^480^K-^603^L
M5	L1	MRP1 ^869^Y-^930^S → MRP2 ^852^F-^925^N
M6	L1-TM12	MRP1 ^931^T-^1013^K → MRP2 ^926^V-^1011^D
M7	TM14-TM15-ICL7	MRP1 ^1085^S-^1190^Y → MRP2 ^1083^D-^1188^F
M8	TM16-TM17	MRP1 ^1191^P-^1270^T → MRP2 ^1189^S-^1268^V

*ABCC1* and chimera genes were cloned into the pcDNA5-FRT vector and expressed in the FlpIn 293 system, allowing a monoclonal expression of each construct, as already done for MRP2^36^. Using the C-terminal MRPm6 epitope 1511-1520, retained in all chimeras, we checked by microscopy and Western blot that the proteins were correctly addressed at the plasma membrane level (Fig. 1b) and their expression efficient (Fig. 1c and Supplementary Fig. S1). The expression was completely impaired for chimeras M1, M4 and M7. M2 and M3 were 50% and 10% produced in respect to the native MRP1 but still correctly addressed as labeled at the plasma membrane level. Finally, M5, M6 and M8 were expressed in a similar way and even better than MRP1 and addressed to the plasma membrane (Fig. 1b,c).

In order to rescue the expression of chimeras M1, M3, M4 and M7, and increase the expression of M2, we tested the effectiveness of the chemical chaperone 4-phenyl butyric acid (4-PBA)^37^, at 5 mM for 24 h in comparison with MRP1 wild type (WT) expression in the same conditions. The 4-PBA treatment rescued chimera M2 but not the four other chimeras (Supplementary Fig. S2a).

**Figure 2:**
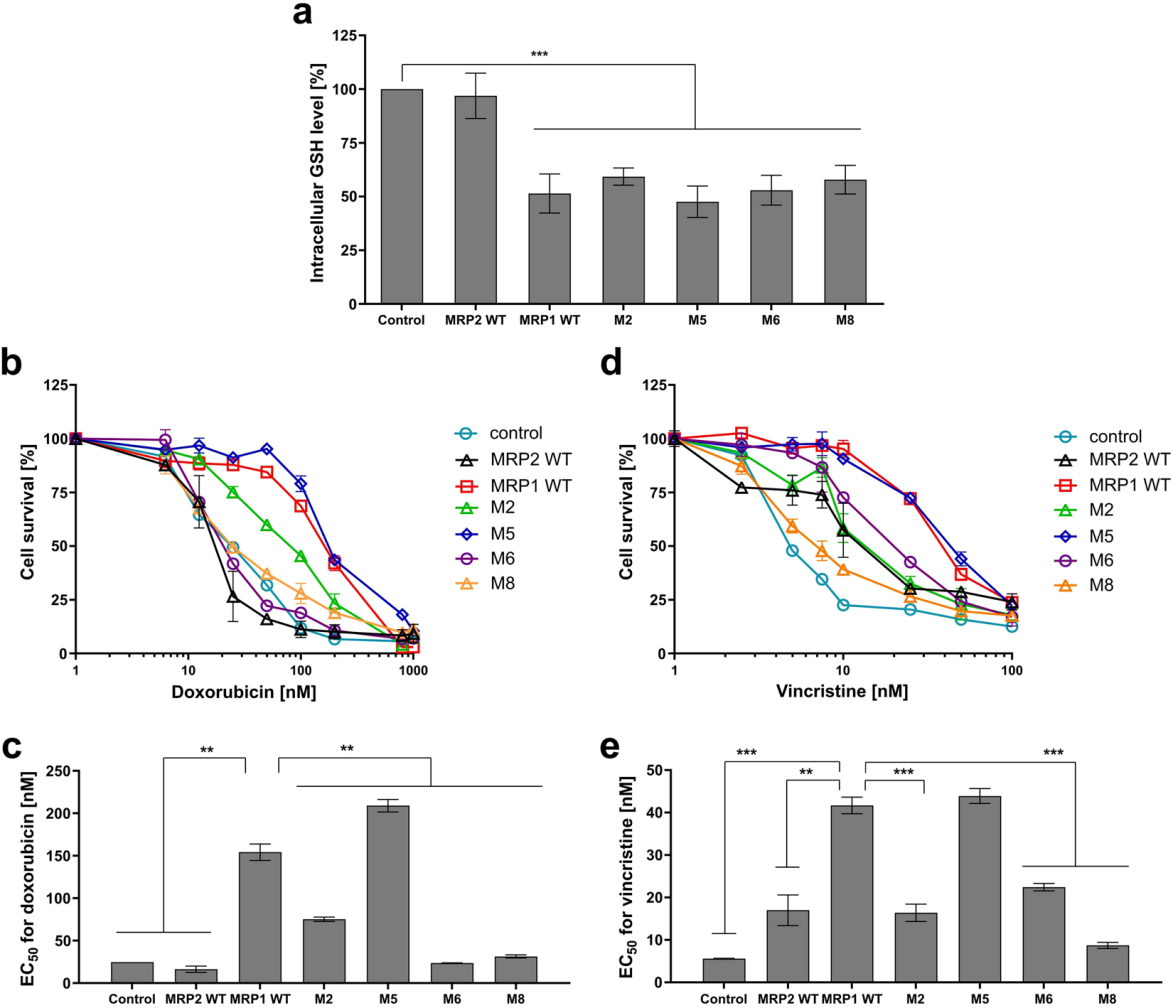
Functionality of MRP1, MRP2 and the different chimera expressed in FlpIn 293 cell line. (**a**) Basal GSH efflux: total intracellular GSH contents were quantified in respect of the same cells transfected with the empty vector (Control, 100%). (**b**-**e**) MDR phenotype: doxorubicin (**b**,**c**) and vincristine cytotoxicity (**d**,**e**) in cells expressing, or not, the different proteins. EC_50_ values are concentrations at which doxorubicin (**c**) and vincristine (**e**) kill 50% of the cell population. Data are the means ± SD of triplicate determinations of N ≥ 3 biologically independent assays. **p ≤ 0.01 and ***p ≤ 0.001 based on a two-tailed paired t test.

In order to probe the correct folding and activity of the expressed proteins, we examined the activity of GSH transport of chimeras compared to that of MRP1 WT and MRP2 wild type (WT). We quantified the amount of glutathione (precisely, GSH + GSSG) in cells expressing MRP1, MRP2 and chimera. Results are shown in Fig. 2a. As expected, GSH level was 2 times lower in FlpIn 293 expressing MRP1 (30 ± 5 nmol/mg protein) than that of the control cells transfected with an empty vector (55 ± 5 nmol/mg protein, taken as 100% intracellular GSH) reflecting the basal GSH transport by MRP1. The GSH content in cells expressing MRP2 was the same than that of the control, whereas MRP2 expression was as expected (Supplementary Fig. S1). This discrepancy is explained by the poor *K*_*M*_ of MRP2 for GSH of about 20 mM^21^, the present concentration of cellular GSH being of 10 mM max. All the cells expressing the different chimera displayed similar GSH levels, in the same range as MRP1 WT. This indicates that the chimeras were well folded at the plasma membrane and that their respective basal transport activity of GSH is not impacted by the switch.

As the transport of substrates and drugs by MRP1 relies on unclear relationships with GSH binding and transport^9,10^, we looked at the ability of the different chimeras to confer a MDR phenotype in a GSH-dependent manner. Hence, we evaluated the cytotoxicity of vincristine (Fig. 2b,c) and doxorubicin (Fig. 2d,e) on the different cell lines in that frame. MRP1 is able, when expressed in the FlpIn 293 system, to transport vincristine and doxorubicin with respective resistance ratios (RR = EC_50_ MRP1-cells/EC_50_ control cells) of 7.26 and 7.13 compared to control cells (Fig. 2b–e respectively). MRP2 transports vincristine with a RR of 3.3 (Fig. 2d,e), slightly higher than that found by Keppler and coll. with MRP2 expressed in HEK293 cells^27^ but is unable to transport doxorubicin (Fig. 2b,c).

The chimera M2 and M5 showed similar or mildly decreased resistance to the drugs compared to MRP1 WT. Chimera M6 and M8, while functional for basal GSH transport activity as shown above, were impacted in their ability to confer resistance to vincristine and doxorubicin (2-fold and 8-fold decrease of resistance to vincristine and doxorubicin respectively for M6, 5-fold and 8-fold decrease of resistance to vincristine and doxorubicin respectively for M8: Fig. 2b–e). Considering the low drug transport activity of MRP2 WT, the M6 was less impacted than M8. The complete loss of drug transport activity of M8 chimera confirms the key roles played by TM16 and TM17 in the drug transport activity of MRP1.

The subsistence of a basal GSH transport despite the disability to confer resistance to drugs co-transported with GSH in chimera M6 and M8 suggests that the two activities can be decoupled, and the transporters remain active. To control that this basal activity is really mediated by MRP1, we mutated in MRP1 WT a cluster of 6 residues that were proven to be involved either in GSH binding or transport, R1138, R1142^38^, S1252, E1253^39^, or in binding of the GSH moiety of LTC_4_, R1249 and N1245^40^. The resulting mutant (all 6 residues were mutated in alanine) was correctly expressed and addressed to the membrane (Supplementary Fig. S3a,b) but the GSH basal transport, as expected, was lost (Supplementary Fig. S3c).

### TM16-TM17 of MRP1 are essential for the stimulated transport of GSH and collateral sensitivity

Since MRP1/MRP2 chimeras were differently impacted in their activities of resistance to drugs co-transported with GSH, we therefore looked at the persistence or disappearance of the activity of the stimulated transport of GSH and collateral sensitivity of the FlpIn 293 expressing MRP1, MRP2 or the chimera to CS agents including verapamil, a well-established CS agent we used as a reference compound. First, we checked that CS agents used in this work, verapamil and 4e, both bind to the transporter. We have previously shown the ability of verapamil to bind to purified human MRP1^11^, indicating it regulates MRP1 by interacting with it. Using another batch of purified protein, kindly gifted by Dr Zachary Lee Johnson and Dr Jue Chen (Laboratory of Membrane Biology and Biophysics, The Rockefeller University, New York, USA), we once again showed that S-verapamil binds MRP1 with a similar affinity of 130 ± 30 nM, in agreement with our previous results^11^. Furthermore, we were able to show the direct binding of the CS agent 4e with a Kd of 78.2 ± 35 nM (Supplementary data S4).

We then recorded the activity of the stimulated transport of GSH mediated by MRP1, MRP2 and chimera. For that, we quantified the intracellular GSH in the presence of verapamil as previously shown in other cell types^8,12^.

**Figure 3:**
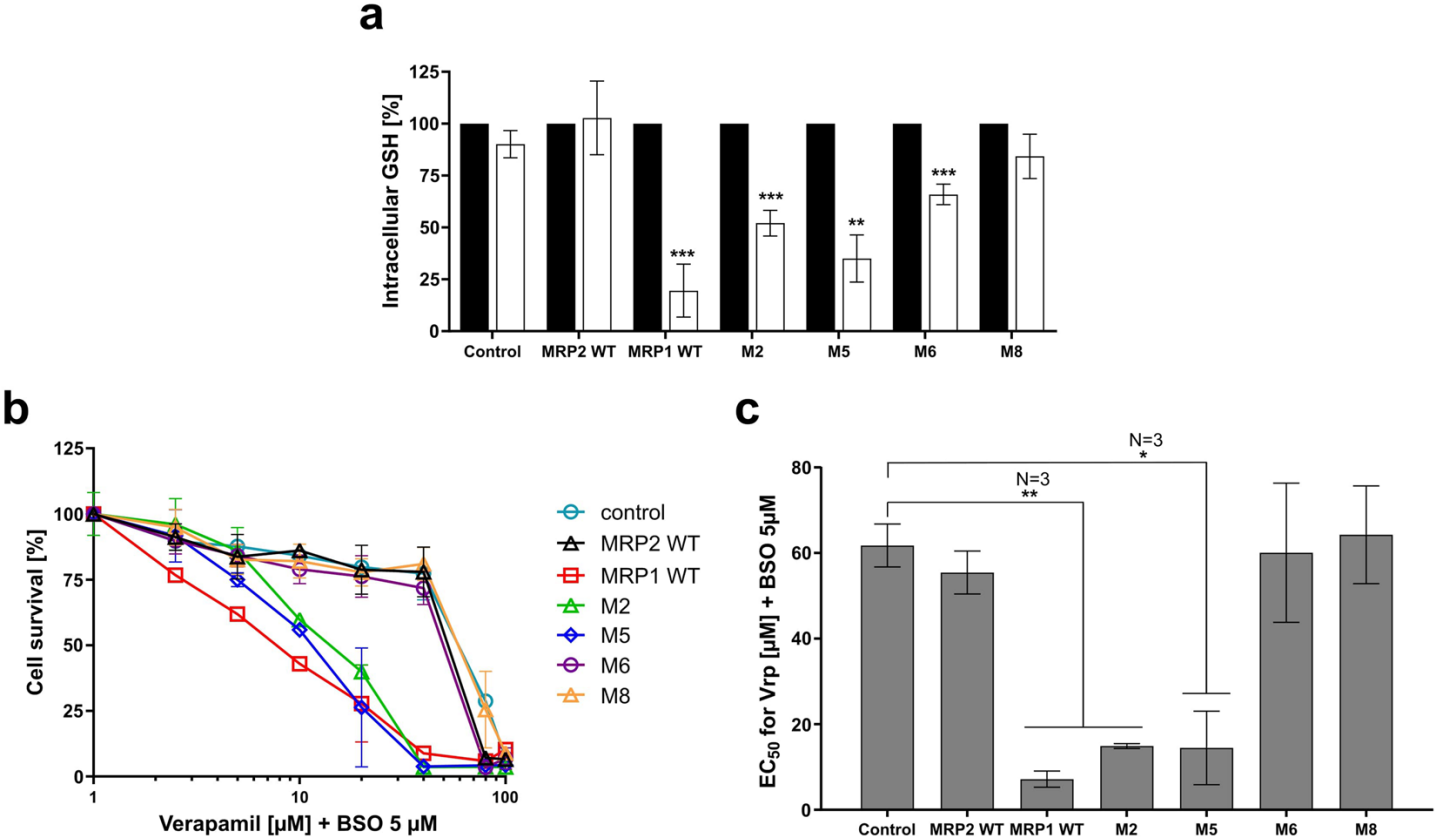
Characterization of collateral sensitivity dependent on MRP1, MRP2, M2, M5, M6 and M8 chimeras. (**a**) Quantification of total intracellular GSH contents in cells in the presence of DMSO (black bars) or verapamil (white bars), in respect of the same cells treated with DMSO as a control (DMSO 100%). (**b**,**c**) CS evaluation after a 72-h incubation with verapamil, in the presence of 5 µM of L-BSO. EC_50_ values are concentrations at which verapamil in presence of L-BSO at 5 µM kill 50% of the cell population. Data are the means ± SD of triplicate determinations of three independent assays. *p ≤ 0,05, **p ≤ 0.01 and ***p ≤ 0.001 (two-tailed paired t test).

MRP1 WT-expressing cells experienced a dramatic GSH level drop of about 80% 3 hours upon addition of 20 µM verapamil. The same treatment had no effect on cells expressing MRP2 as already shown when MRP2 was expressed in canine kidney cells^24^ (Fig. 3a, white bars). Compared to that observed in cells expressing MRP1, the stimulation of GSH efflux in the presence of verapamil was still maintained with M5, half-reduced with M2, more strongly impacted with M6 and almost completely lost with M8 (Fig. 3a, white bars). It is interesting to note that the mutation in alanine of the 6 residues involved either in GSH binding or transport, in addition to the loss of GSH basal transport previously observed (Supplementary Fig. S3c), led also to the loss of the GSH efflux stimulation by CS agents (Supplementary Fig. S3d).

To investigate whether the low protein expression of M2 is causing this reduction in GSH efflux stimulation, the amount of intracellular GSH was measured in cells rescued or not for chimera M2 using the chemical chaperone 4PBA. As shown in Supplementary Fig. S2b, the effect of verapamil on GSH level did not increase after 4-PBA treatment. These results indicate that despite enhancing protein expression in cells expressing chimera M2 (Supplementary Fig. S2a), the stimulation of GSH transport by verapamil does not change, as also observed for MRP1. A higher level of protein expression does not necessarily improve the GSH efflux stimulation.

We then looked at the cytotoxicity of verapamil for MRP1 or MRP2 or chimera-expressing FlpIn 293 (Fig. 3b,c). We previously described that the verapamil stimulated GSH efflux mediated by MRP1 triggers cell death of Baby Hamster Kidney cells overexpressing MRP1 after transfection with the *MRP1* gene and in human small cell lung cancer H69AR cells overexpressing MRP1 after doxorubicin selection^12,15^. Verapamil alone cannot trigger CS in MRP1 expressing FlpIn 293 (Supplementary Fig. S5b, closed squares); this lack of effect could be related to the quite low expression level of MRP1 because of the presence of a single copy of the *MRP1* gene in the genome of MRP1-expressing FlpIn 293 cells. We added 5 µM L-BSO (L-buthionine sulfoximine), an inhibitor of the *de novo* synthesis of GSH. The BSO is by itself a CS agent for MRP1-expressing FlpIn 293 (Supplementary Fig. S5a), as already shown for other MRP1-expressing cell lines^41,42^. At a low, not toxic, concentration of 5 µM (i.e. Supplementary Fig. S5a), BSO potentiates the cytotoxic effect of verapamil in cells overexpressing MRP1 (Supplementary Fig. S5b, open squares).

Cells expressing MRP1 were hypersensitive to verapamil mixed with L-BSO while cells expressing MRP2 showed a sensitivity similar to control cells (Fig. 3b,c), in correlation with the behavior of the corresponding cell lines with respect to the GSH efflux stimulation (Fig. 3a). We then looked at the cytotoxicity of verapamil on the other cell lines in presence of L-BSO (Fig. 3b,c). Cells expressing chimera M2 and M5 remained nearly as sensitive as MRP1 WT to verapamil mixed with L-BSO. Cells expressing chimera M6 and chimera M8 showed an 8.5 to 9-fold (EC_50_ of 60 µM and 64 µM respectively, compared with 7 µM for MRP1 WT) decrease of verapamil + L-BSO sensitivity (Fig. 3b,c). These data correlate the behavior of the corresponding cell lines with respect to the GSH efflux stimulation observed above in the presence of verapamil (Fig. 3a).

### The modeling of TM16-TM17 allows the identification of putative residues for the stimulated transport of GSH

**Figure 4:**
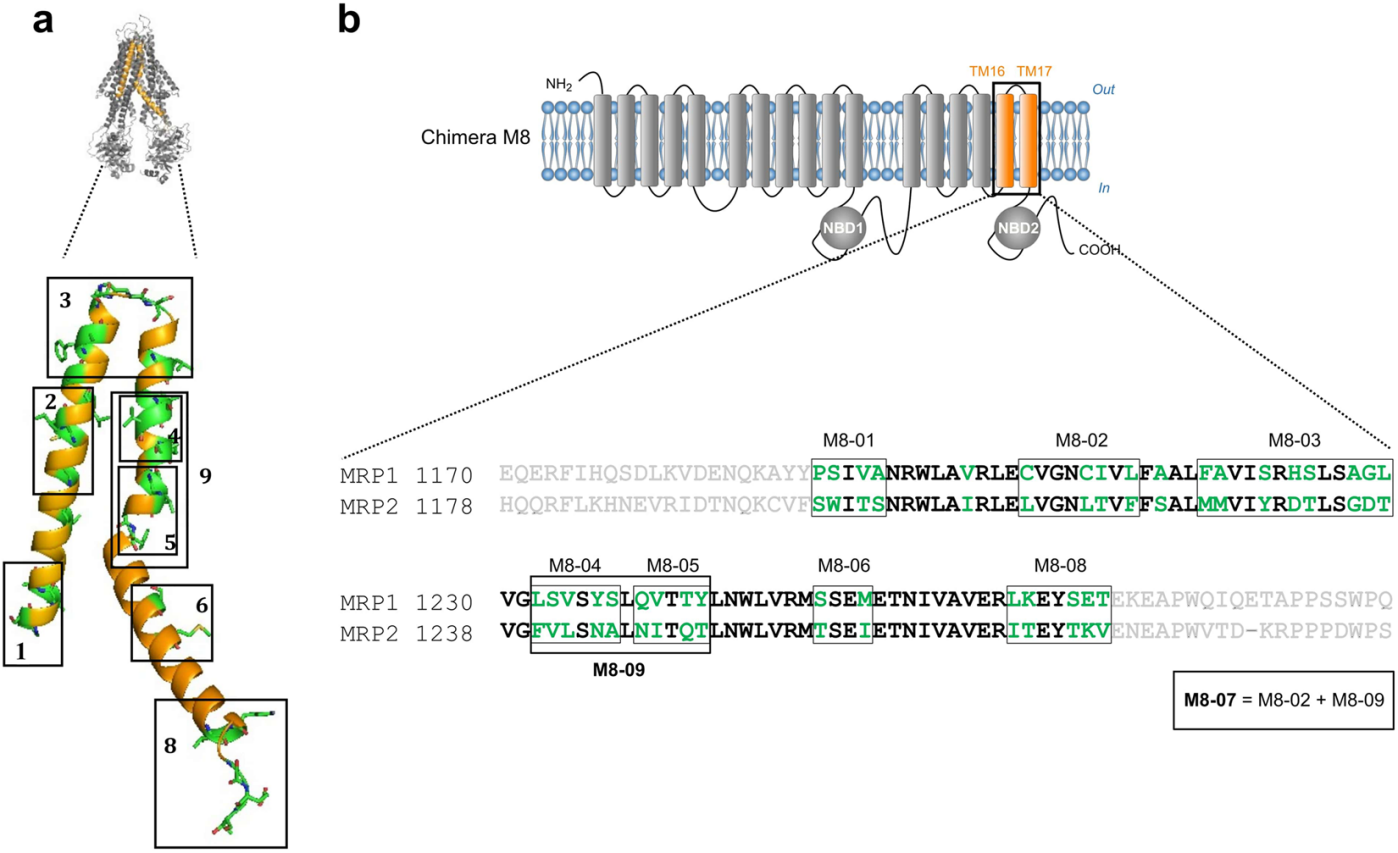
Processing chimera M8 mutants. (**a**) Cartoon representation of MRP1 model based on Na-ATM1 3D structure. The orange colored region represents the MRP2 fragment in the chimera M8. MRP2 residues that differ from MRP1 residues are colored in green. The mutants are shown in black boxes. (**b**) Amino acid alignment of MRP1 and MRP2 in the modified region of chimera M8 (represented in bold). MRP2 residues that differ from MRP1 residues are colored in green. All nine mutants are shown in black boxes.

The above results led us to conclude that among the expressed chimeras, M8 is the most significantly affected both for inducing CS and for its GSH-dependent transport activity. We therefore focused on the swapped region encompassing residues 1191 to 1270 forming the TM helices 16 and 17. Within the region replaced in chimera M8, 34 amino acids are different between MRP1 and MRP2. We partially restored the original residues of MRP1 in the MRP2 segment of M8 in order to recover the GSH efflux stimulation activity. In order to better grasp the degree of residue modification in chimera M8, we built a homology model to serve as a 3D base for further amino-acid substitution (Fig. 4a). As a model, we used the crystal structure of *Na*Atm1, an MRP1 ortholog from *Novosphingobium aromaticivorans*^43^. This transporter was crystallized with either GSH (PDB code **4MRP**), or GSSG (PDB code **4MRS**) at high enough resolutions (2.5 and 2.35 Å, respectively) to precisely locate each substrate, and the structure was solved using selenomethionines that ascertained sequence assignment. This model of MRP1, built long before the release of the Cryo-EM structure of human MRP1^40^, remains in adequacy with the published Cryo-EM structure, validating our choice of mutants. Based on this model, we designed nine mutants (M8-01 to M8-09) (Fig. 4b).

### Glycine 1228 is the sole residue responsible for the stimulation of GSH transport and associated collateral sensitivity in TM16-TM17

**Figure 5:**
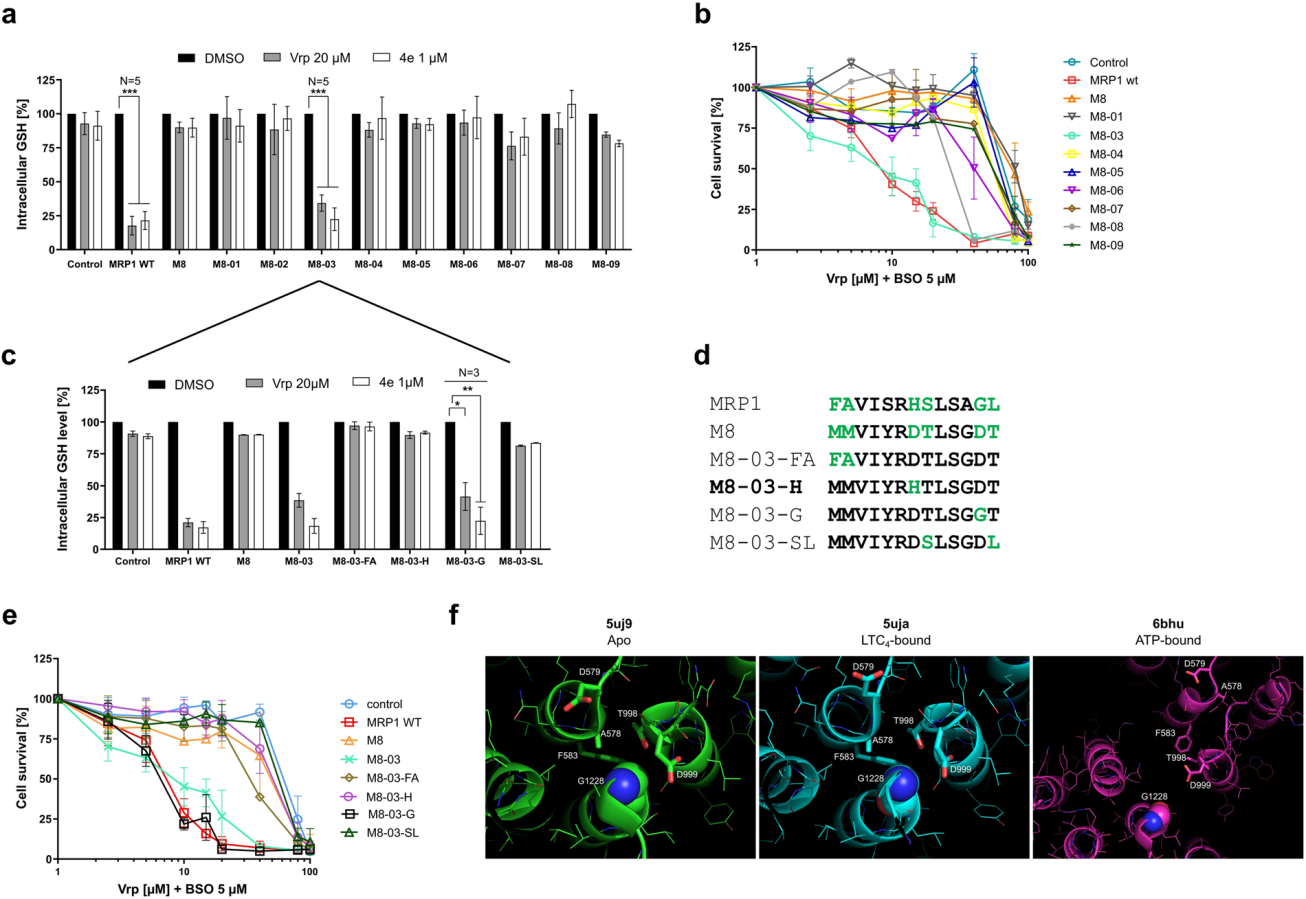
Intracellular GSH content and collateral sensitivity of cells overexpressing chimera M8 and its mutants. (**a**,**c**) Quantification of the intracellular GSH contents after 3 h incubation with 1 µM of 4e or 20 µM of verapamil. (**b**,**e**) CS evaluation in cells expressing the different proteins after a 72-h incubation with verapamil, in the presence of 5 µM of L-BSO. (**d**) Detailed sequences of each M8-03 mutant. (**f**) Close-up view of the structures surrounding of G1228. The number displayed on the images correspond to human MRP1. G1228 is displayed as spheres. Close and relevant residues are displayed as sticks. Data are the means ± SD of triplicate determinations of three independent assays. ***p ≤ 0.001 (two-tailed paired t test).

To characterize the effect of the original replacement within the nine identified clusters of chimera M8, we first verified that all the mutants were well expressed and addressed to the plasma membrane (Supplementary Fig. S6a,b). Among the 9 constructs, only M8-03 restored the stimulation of GSH efflux by verapamil, confirmed by the other, very potent, CS agent 4e^18^ (Fig. 5a). In line with this result, collateral sensitivity was only restored in the cells overexpressing this mutant (Fig. 5b).

**Figure 6:**
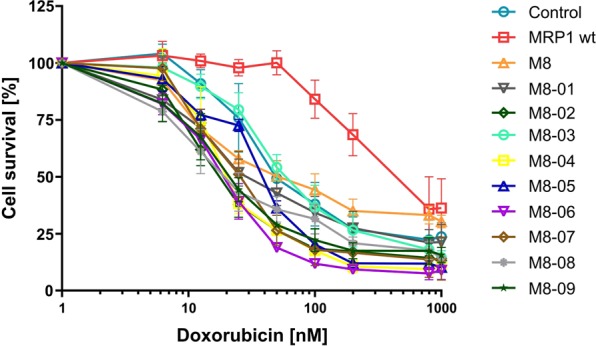
MDR phenotype characterization of FlpIn 293 cells overexpressing the mutants. Cytotoxicity to doxorubicin. Data are the means ± SD of three independent assays.

However, similar to the original chimera M8, chimera M8-03 failed to recover the GSH-dependent resistance to drugs like doxorubicin (Fig. 6).

We then sub-clustered the residue replacement by generating four additional mutants of M8-03, as shown in Fig. 5d: M1217F + M1218A leading to the M8-03-FA cell line, D1223H (M8-03-H), D1228G (M8-03-G), T1224S + T1229L (M8-03-SL). S1221Y and A1227G were not considered because of the relative similarity of the amino acids. The protein expression levels (Supplementary Fig. S7a) and the intracellular GSH content (Supplementary Fig. S7b) in cells overexpressing these mutants were quite similar to the cells expressing wild-type MRP1.

We then measured the intracellular GSH content in the presence of the two CS agents, verapamil or 4e. Remarkably, the sole restoration of the glycine 1228 residue in the M8-03 construct led to the recovery of GSH-efflux stimulation initially lost in M8 (Fig. 5c). Moreover, cells expressing the mutant M8-03-G were hypersensitive to verapamil + L-BSO, similarly to wild-type MRP1 and mutant M8-03 cells (Fig. 5e). In contrast, no significant cytotoxicity was observed in the other cell lines showing that in chimera M8-03, the phenotype of MRP1-induced cell-death is linked to a single amino-acid substitution, D1228G. The four M8-03 mutants were unable to restore the doxorubicin resistance observed in FlpIn 293 cells overexpressing wild-type MRP1 (Supplementary Fig. S7c). The restoration of this single glycine residue is thus sufficient to recover the GSH efflux stimulation, but not the drug transport, which further suggests a different mechanism for these two transport activities.

To evaluate the structural impact of G1228D mutation, we visualized the G1228 in the three Cryo-EM structures of bovine MRP1^40,44^, the apo, LTC_4_-bound and ATP-bound structures (Fig. 5f). The sequence identity between human and bovine MRP1 is 91%, all residues forming the extracellular loops are conserved between the two species. Considering the position of the Gly 1228, we could argue that the mutation G1228D will trigger a steric hindrance with T998 that is tightly constrained by F583 and cannot move easily in the apo and LTC_4_-bound conformations. In the ATP-bound conformation, the mutation of Gly to Asp will have limited expected effects, resulting most probably in a rotamer rearrangement in respect of D999 with limited consequences on the backbone position.

## Discussion

MRP1 is overexpressed in many types of resistant cancer. Patients with neuroblastoma expressing MRP1 have a poor prognosis^3^. Recently, its expression was shown to be correlated with a bad prognosis in the pathogenetic process of colorectal cancer^45^. One of the most promising strategies to overcome this multi-drug resistance, MRP1-mediated CS, relies on the ability of certain ligands of MRP1 to induce a massive MRP1-dependent GSH efflux, that depletes the cells of their anti-oxidant defense, leading to their selective cell death^15^. The molecular mechanism leading to such a phenomenon is difficult to understand. Therefore, identifying the structural determinants of the ligand-stimulated GSH transport in MRP1, and evaluating its relationship with the basal transport of GSH and the transport of the drug, constitutes one central yet unknown cornerstone to be able to propose MRP1-mediated CS as an anti-cancer therapy. To address this question, we generated 8 different MRP1/MRP2 chimeras and characterized their ability to transport GSH in a stimulated manner and to confer drug resistance. The functional characterization of these chimeric proteins constituted a challenge considering the poor or even none expression of some of the constructs. This lack of expression can be explained in light of previous mutagenesis studies which showed that some residues or sequences encompassed by TM5-L0 (chimera M1), ICL5-TM10-TM11 (chimera M4), TM14-TM15-ICL7 (chimera M7) are important for the correct addressing and folding of MRP1^19,34,46,47^. Bandler *et al*. notably described that the substitution of the first 290 residues of MRP1 by the corresponding residues of MRP2, like in chimera M1, leads to a complete absence of addressing to the plasma membrane with a retention in the endoplasmic reticulum^19^. Although most of the residues in those regions were identical or very similar between MRP1 and MRP2, synonymous mutations or difference in the choice of codons can result in serious protein misfolding^48^. Up-to-now there is no evidence in literature referring to the substitution of residues that could explain an impairment of expression or addressing for chimera M3, covering TM6-TM7.

Among the four expressed chimeras, M2 (the C-terminus of L0) and M5 (N-terminus half of L1), kept to a certain extent their ability to transport GSH in a stimulated manner and consequently to sensitize cells to GSH-depleting agents despite their relatively low expression compared to wild-type MRP1. Cells expressing M2 exhibited a CS considerably high, despite the decrease of the GSH efflux stimulation (Fig. 3a). The latter is however sufficient to lead to a substantial CS in presence of verapamil as already shown with other potent CS agents able to induce a strong CS while GSH efflux stimulation ranged between 34 to 78%^18^. It should be noted that compounds that stimulate the higher GSH efflux are not always the best to induce CS and conversely. For instance, we showed that the flavonoid galangin triggers a strong MRP1 mediated GSH efflux without inducing cell death. Therefore, we previously suggested that “GSH efflux is necessary to some extent, but not sufficient, for the selective death induction of MRP1-expressing cells”^18^.

M6 (second part of L1 and TM12) and M8 (TM16-TM17), while retaining the ability to transport GSH, were greatly impaired, mainly for M8, in their ability to transport it in a stimulated manner and consequently to sensitize cells to GSH-depleting agents. The results obtained with the chimera M8 and its mutants allowed the identification of a key residue in the extracellular loop between TM16 and TM17, the G1228 residue of MRP1. When restored, this residue allows chimera M8 to only regain the ability of a GSH efflux stimulation by CS agents, thereby inducing collateral sensitivity, but not the ability to confer drug resistance. This result confirms that the mechanism of binding and transporting drugs by MRP1 is different from the mechanism required for the stimulation of GSH efflux by verapamil. This was also observed by Ito *et al*., who showed that W1246C MRP1 mutant (TM17) lost the ability to confer resistance to vincristine, doxorubicin, and etoposide, while its ability to stimulate a GSH transport in the presence of verapamil remained intact^49^. Furthermore, if GSH efflux stimulation was abolished with the G1228D mutant, the basal GSH transport was not, suggesting also that the mechanism of stimulated GSH transport is different from the non-stimulated one.

**Figure 7:**
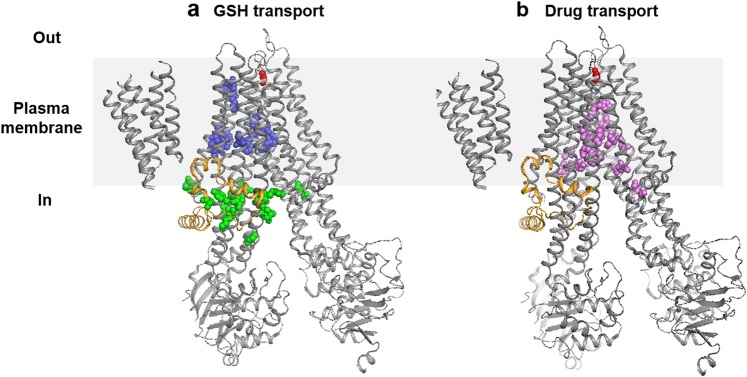
Ribbon diagram illustrating the 3D structure of MRP1 determined by Johnson, Z.L. *et al*. (47). The approximate position of the membrane is represented by a grey rectangle separating the periplasm (Out) and the cytoplasm (In). The L0 domain is represented in orange and the amino acid G1228 is represented in red spheres. (**a**) Location of the amino acids shown to be implicated in GSH transport that could form the GSH cytoplasmic site (green spheres: zone G1) and the GSH membrane site (blue spheres: zone G2). (**b**) Location of the amino acids shown to be implicated in vincristine, doxorubicin and etoposide transport that could form the drug membrane site (purple spheres: zone D).

To investigate the mechanism of GSH dependent transport mediated by MRP1, we localized on the 3D structure of the transporter^40^ two putative GSH binding sites deduced from the numerous mutagenesis studies done in the past two decades on MRP1 compiled in the database http://abcmutations.hegelab.org ^50^, one located in the cytoplasmic face and the other located in the membrane (Fig. 7a, zones G1 and G2).

The existence of these two GSH binding sites is also corroborated by a George’s team study that used a radiolabeled photoactive analog of GSH (IAAGSH)^31^. Additionally, a potential drug binding site can also be predicted based on previous mutagenesis studies that allowed the identification of residues implicated in the transport of doxorubicin, vincristine, and etoposide. We pinpointed this site on the 3D structure of MRP1 (Fig. 7b, zone D). It should be noted that the G1228 is located far away from all 3 sites, (D, G1 and G2), validating the independence of the mechanism of GSH efflux stimulation relying on G1228 from the basal GSH transport and GSH-dependent transport of drugs. To explain these two last transport activities, we propose that the first cytoplasmic group of amino acids associated with GSH transport (zone G1 Fig. 7a) can form a vestibule that could serve as the entry gate of GSH into the transporter from the cytoplasm. A GSH molecule could probably bind from the cytoplasm to zone G1, from which the L0 motif then translocates this molecule to zone G2, to be transported out of the cell. The L0 motif was indeed shown to be essential to the photolabeling of each moiety of MRP1 by the azidophenacyl-[^35^S]GSH whereas it was not itself photolabeled^51^. The hydrophobic drugs might interact with zone D of the transporter directly from the lipid bilayer, in a process called vacuum cleaner^52^. As mentioned before, these drugs are co-transported with GSH. Importantly, photolabeling studies using the tricyclic isoxazole photoaffinity label [^125^I]LY475776 suggested that GSH binding on MRP1 is a prerequisite for binding of LY475776 and so comes prior to the drug binding^51^. Going back to our model, this could mean that the GSH binds within the zone G2, which causes a conformational change that allows the binding and the transport of the drug by zone D, which is followed by the release of GSH.

One central remaining question would be how the amino acid G1228 can be implicated in GSH efflux stimulation while it is not located in or near GSH binding sites? First, we could argue that the identified G1228 is not localized in the binding site of verapamil. Several previous results seem to indicate that verapamil binds to the drug binding site. Indeed, vincristine and verapamil both were shown to decrease the photolabeling of MRP1 by [^125^I] LY475776, an inhibitor of LTC_4_ transport^53^. Wong *et al*. also showed that the dimer of flavonoid 4e, used here as CS agent, is a competitive inhibitor of doxorubicin^54^. Furthermore, we have shown that verapamil and its iodinated derivative NMeOHI_2_ inhibited leukotriene C_4_ transport by MRP1-enriched membrane vesicles in a competitive manner^12^.

Verapamil and drugs such as vincristine however, when bound in zone D, probably interact with MRP1 in different ways, as already suggested^8^ and stimulate GSH uptake also in different ways. Verapamil could probably induce a specific conformational change where the G1228 plays an important role. In the recent publication presenting the CFTR (Cystic Fibrosis Transmembrane conductance Regulator) 3D structure, also a member of the ABCC family as ABCC7, the authors described a possible tunnel gate region formed by residues from extracellular ends of TM 1, 6, 8 and 12 which are equivalent to TM 6, 11, 13 and 17 in MRP1 (ABCC1). They suggested that a conformational change of these helices could open the gate and form a large ion channel inside the hydrophobic plasma membrane^55^. Such a gate could exist in MRP1 and G1228 could be an essential element for its functioning.

On another hand, CFTR has been described as a “broken” transporter because it is an ion channel that has evolved from an ABC transporter. A lateral portal, unique to CFTR, by which the cytoplasmic Cl− ions probably enter the pore CFTR, cracked open between TM helices 4 and 6 in the NBD-dimerized conformation^56^. MRP1 shows a cytoplasmic GSH site (we called G1) that we could consider as a lateral portal. Could MRP1, under the action of verapamil, become a “broken” transporter adopting a conformation “channel like” too? The strong GSH efflux induced by verapamil is energy dependent since MRP1 K1333L, a mutant with a defective ATPase activity, is unable to confer CS in cells overexpressing it^12^. Additionally the GSH uptake stimulated by verapamil was already proved to be ATP-dependent^8^. However, MRP1 is unlikely to adopt the ATP-dependent transition from inward to outward conformation to transport a single molecule of GSH, because if such was the case, a strong stimulated GSH efflux would require the hydrolyzation of a large number of ATP, and this would not comply with the very low ATPase activity of MRP1^57^. In agreement with this hypothesis, Cole *et al*. in 2000^8^ mentioned some unpublished data showing that verapamil does not stimulate MRP1 ATPase activity(+/−GSH), while it is known at being a potent stimulator of the P-glycoprotein ATPase activity. These experiments were conducted on purified MRP1 proteins reconstituted in membranes. Like CFTR^56^, it would be tempting to think that NBDs dimerization and ATP hydrolysis would drive MRP1 “channel gating”, after verapamil binding, with ATP hydrolysis being necessary for the opening and closure of the pore. We formulate the hypothesis that CS agents, like verapamil, could induce an important conformational change on MRP1 resulting in a continuous translocation of GSH through a gate opened by the binding of verapamil. The presence of the D1236 (from MRP2 substitution) instead of G1228 (wild-type MRP1) in the chimera M8 could create a steric hindrance, as shown by the Cryo-EM apo and LTC_4_ bound (inward-facing) structures of bovine MRP1^40,44^ (Fig. 5f), that could block the opening of this GSH gate. This residue G1228 is located in a critical region of the molecule in close proximity to the amino acid F583 (see Fig. 5f), shown to be crucial for signaling between the membrane-spanning and nucleotide-binding domains^58^.

If we cannot rule out that other important putative residues in the areas covered by the chimeras M1, M3, M4 and M7, not expressed at the plasma membrane, could play a role in CS, we were able to identify the Glycine 1228 as a key residue for the stimulation of GSH efflux and CS, possibly located in a GSH gate. This Glycine 1228 is discriminating the activity of the stimulated transport of GSH from the other transport activities mediated by MRP1.

## Materials and Methods

### Cell lines

FlpIn 293 cells (Life Technologies) with low passage number were transfected with empty pcDNA5/FRT vector or pcDNA5/FRT vector containing either wild-type MRP1, wild-type MRP2, or MRP1/MRP2 chimeras and mutants. These vectors were co-transfected with the pOG44 vector according to Life Technologies. This co-transfection resulted in the targeted integration of the expression vector to the same locus in each cell resulting in isogenic cell lines. Transfected cells were grown at 37 °C in 5% CO_2_ in DMEM high glucose GlutaMAX (Gibco-Life Technologies) supplemented with 1% penicillin/streptomycin (Sigma Aldrich) and 5% of heat-inactivated fetal bovine serum (PAA, GE Healthcare Life Sciences) and selected with 0.2 mg. mL^−1^ of Hygromycin B (Life Technologies) for 2 weeks. Phenotyping tests were conducted by observation of characteristic cell morphology, drug responsiveness and expression of MRP1. Passage number of cell lines is between 2-3 times a week for a maximum of 4 weeks. All cell lines were tested for Mycoplasma contamination (Mycoalert Mycoplasma Detection Kit (Lonza).

### Generation of MRP1 and MRP2 vectors

*ABCC1* gene was subcloned from a previous pcDNA3.1(-)-MRP1, using the restriction enzyme XbaI and NotI to extract the *ABCC1* insert. The insert included both an upstream Kozak sequence (G/A)NNATGG (where N = T or C) to promote the initiation of the translation and a downstream poly-histidine tag (10-His) to allow Ni-agarose affinity purification of the protein product. Correct subcloning of *ABCC1* insert was attested by Nhe I and Not I digestion profile of the vectors and sequencing of the vector. The MRP2 expression vector pcDNA5/FRT-MRP2 was constructed by cloning the *ABCC2* gene into the eukaryotic expression vector pcDNA5/FRT (Life Technologies) as described previously^59^ with an additional Kozak sequence added at the beginning of the gene.

### MRP1/MRP2 chimera’s construction

A couple of unique restriction enzyme sites x and y in the *ABCC1* gene of the pcDNA5/FRT-MRP1 vector were selected to surround a fragment A which includes the fragment B, corresponding to the sequence of *ABCC1* to exchange by the sequence of *ABCC2* (Supplementary Table 1).

The cDNA fragments A containing the sequence B from *ABCC2* and the restriction sites surrounding them were synthesized by the Eurogentec company and inserted into a shuttle vector. The pcDNA5/FRT-MRP1 was digested with restriction enzymes x and y and ligated (blunt-end ligation) with the digested fragment A to obtain the vector expressing the chimera MRP1/MRP2. The fidelity of the constructs was checked by both sequencing and restriction enzyme analysis. Exchanged amino-acid fragments are shown in Table 1.

### Chimera M8 mutants construction

The constructs were generated by the same method described for the chimeric constructs. Several fragments A of the chimera M8 (Supplementary Table 1) were synthesized by the Eurogentec company with the replacement of different clusters of MRP2 amino acids (detailed in the Fig. 4 for each mutant) by MRP1 amino acids. The same strategy was used for the construction of the single mutants of the chimera M8 (detailed in Fig. 5d).

### Western blot analysis

The analysis was performed as previously described^18^. The membranes were probed with either the anti-MRP1 monoclonal antibody MRPm6 or the anti-MRP2 monoclonal antibody M2I-4 (Alexis Biochemical), and a polyclonal alpha-tubulin antibody as loading control (Sigma Aldrich).

### Immunofluorescence confocal laser microscopy

1.10^5^ cells were cultured at 37 °C in 5% CO_2_ for 48 h in a polystyrene vessel chamber on tissue culture treated glass slide (BD Falcon). Cells were fixed with 4% paraformaldehyde in PBS for 30 min and washed with PBS Ca^2+^/Mg^2+^ (Sigma-Aldrich). Cells were then permeabilized with 0.1% Triton-X-100 (Sigma-Aldrich) for 5 minutes, then incubated for 1 h with a saturation solution (10% of goat serum (Sigma-Aldrich), 3% of human serum (Sigma-Aldrich), 0.1% Triton-X-100 (Sigma-Aldrich), 1% BSA (Euromedex), 0.05% Tween 20 (Euromedex) in PBS. Cells were then incubated at 4 °C overnight with the anti-MRP1 monoclonal antibody MRPm6 (Alexis Biochemical), washed and then incubated for 1 h with goat secondary antibody anti-mouse IgG coupled to Alexa Fluor 488 (Life Technologies). Nuclei were counterstained with 4 µM of Hoechst 33258 for 10 min. Samples were examined under a Compact Confocal Power Pack (ZEISS LSM 800 with Airyscan) with a 40 (numerical aperture, 1.2) oil immersion lens. The magnification used may be different depending on the samples.

### Cell survival analysis by MTT assay

Cells were plated in 96-well plates at 2.0 × 10^4^ cells/well for all cell lines. Cells were allowed to attach overnight before the addition of compounds solubilized in dimethylsulfoximide (DMSO) and diluted in complete culture medium with a fixed final DMSO concentration of 0.5%. Cells were then incubated for 72 h at 37 °C under 5% CO_2_. A solution of MTT at a final concentration of 0.5 mg. mL^-1^ was then added and 3 to 4 h hours later, the medium was aspirated, and the formazan product was solubilized by the addition of a ethanol/DMSO (1:1) solution. Optical density (OD) was measured on an OD plate reader using a reference wavelength of 690 nm and a test wavelength of 570 nm. OD 570-690 obtained in the control well containing DMSO treated cells is considered as 100% of survival.

### Total cellular glutathione determination

Total cellular glutathione content was measured using the enzymatic method described by Tietze and adapted by Anderson^60^ to the Microtiter plate. Cells (1.0 × 10^5^ cells/well into 96-well plates) were allowed to attach overnight, in the presence or absence of 5 mM 4-PBA, followed by incubation for 3 hours with CS agents at different concentrations. The DMSO concentration was set at 0.5%. Each condition was run in triplicate. Total intracellular glutathione contents and protein titration were then assessed as previously described^61^.

### Measurement of binding of CS agents on MRP1

The impact of ligand binding on intrinsic fluorescence was monitored using a Xenius XC spectrofluorometer (SAFAS) in quartz cuvettes containing 200 µl of MRP1 at 0.5 µM in 150 mM KCl, 50 mM Tris pH 8.0, 2 mM MgCl_2_, 2 mM DTT, and 0.06% digitonin. Titration of S-verapamil (Sigma-Aldrich) and ligand 4e (synthesized as previously reported^62^) was carried out by 2 µl additions of ligands solubilized in 50% DMSO. This yielded a final DMSO concentration of 4%, which was previously shown to not affect MRP1’s structure^40^. Tryptophans were excited at 295 nm and emission was monitored between 310 and 380 nm, with a 2 nm step, 0.3 s acquisition average, 5 nm excitation bandwidth and 3 nm for emission. Data were acquired in triplicates for MRP1, subtracted from the buffer fluorescence. F/Fo for MRP1 was displayed as a function of ligand concentration. Data were fitted in Prism 7 (Graphpad) using one site specific binding with a non-specific contribution. Y = B_max_*X/(Kd + X) + NS*X + Background (X: ligand concentration, Y: total binding, B_max_: Maximum binding, Kd: Equilibrium dissociation constant, NS: slope of the nonlinear regression, Background: measured binding with no added ligand).

### Homology modeling

The homology model was made to establish secondary chimeras and to visualize the effects of mutations. The model of the glutathione transporter from *Novosphingobium aramaticivorans Na*Atm1^43^ (4MRS) was chosen as it was solved at high resolution (2.5 and 2.35 Å) and with selenomethonines to ascertain the registry of the structure. In addition, this structure was solved apo or in complex with glutathion allowing a verification of the residues of human MRP1 liganding the substrate. A sequence alignment with the N- and C-terminal halves of human MRP1 was performed using the AlignMe software in slow mode to take into account the trans-membrane region in the alignment. Alignments were then manually optimized with the rationale to avoid insertion/deletion within trans-membrane fragments, but rather move them into loops, and to anchor the beginning or the end of TM helices on special residues, common to the two proteins, like for instance, positively charged residues inside, proline to introduce kinks in helices, etc. The sequence of human MRP1 was then threaded on the structure of Na-Atm1 using default parameters of the program MODELLER^63^. The threaded structure was then manually inspected using Coot^64^ and energy-minimized using REFMAC^65^. The model was validated in Coot showing that >95% of the residues reside in the most favorable region of the Ramachandran plot.

### Statistical analysis

Statistical analysis was performed for each experiment on N ≥ 3 biological independent assays using a two-tailed paired t test with unequal variance. *p < 0.05, **p < 0.01, ***p < 0.001 were used as symbols to represent significance levels. Standard Deviations (SD) to quantify the dispersion of sets of data to the mean were calculated as indicated in each figure legend.

## Supplementary information

Supplementary information.
