# Supplementary material for: Molecular analysis of the massive GSH transport mechanism mediated by the human Multidrug Resistant Protein 1/ABCC1
Source: Sci Rep. 2020 May 6;10:7616. doi: 10.1038/s41598-020-64400-x (PMC7203140; doi:10.1038/s41598-020-64400-x)
Supplement: Supplementary file 1 — Supplementary information. [file 41598_2020_64400_MOESM1_ESM.pdf]

Supplementary information

TITLE: Molecular analysis of the massive GSH transport mechanism mediated by the human Multidrug Resistant Protein 1/ABCC1

AUTHORS: Rachad Nasr, Doriane Lorendeau, Ruttiros Khonkarn, Lauriane Dury, Basile Pérès, Ahcène Boumendjel, Jean-Claude Cortay, Pierre Falson, Vincent Chaptal, Hélène Baubichon-Cortay

Supplementary Table 1

Exchanged nucleotide fragments in MRP1 with the corresponding MRP2 fragments for MRP1/MRP2 chimeras constructs. The fragment A includes the fragment B corresponding to the sequence of MRP1 to exchange by the sequence of MRP2.

Chimera name	Exchanged Nucleotide Fragment B	MRP1 limits of Nucleotide Fragment A	Restriction enzymes
M1	MRP1 ^{501}G-^{839}G → MRP2 ^{483}A-^{800}A	1 - 846	Nhe1/BamH1
M2	MRP1 ^{846}G-^{947}G → MRP2 ^{807}G-^{908}T	846 - 1901	BamH1/Bsu36I
M3	MRP1 ^{948}T-^{1187}G → MRP2 ^{909}C-^{1148}G	846 - 1871	BamH1/Bsu36I
M4	MRP1 ^{1476}G-^{1847}G→ MRP2 ^{1437}A-^{1808}G	846 - 1871	BamH1/Bsu36I
M5	MRP1 ^{2604}T-^{2789}C→ MRP2 ^{2553}T-^{2774}T	1871 - 3273	Bsu36I/SexA1
M6	MRP1 ^{2790}A-^{3038}A→ MRP2 ^{2775}G-^{3032}C	1871 - 3246	Bsu36I/SexA1
M7	MRP1 ^{3252}T-^{3569}C→ MRP2 ^{3246}G-^{3563}T	3237 - 4083	SexA1/Cla1
M8	MRP1 ^{3570}C-^{3809}T → MRP2 ^{3564}T-^{3803}G	3237 - 4083	SexA1/Cla1

Supplementary Figure S1

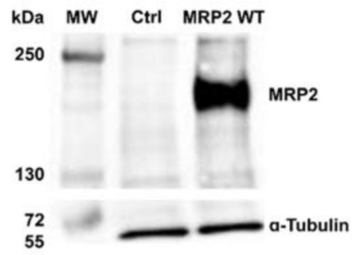

Supplementary Figure S2

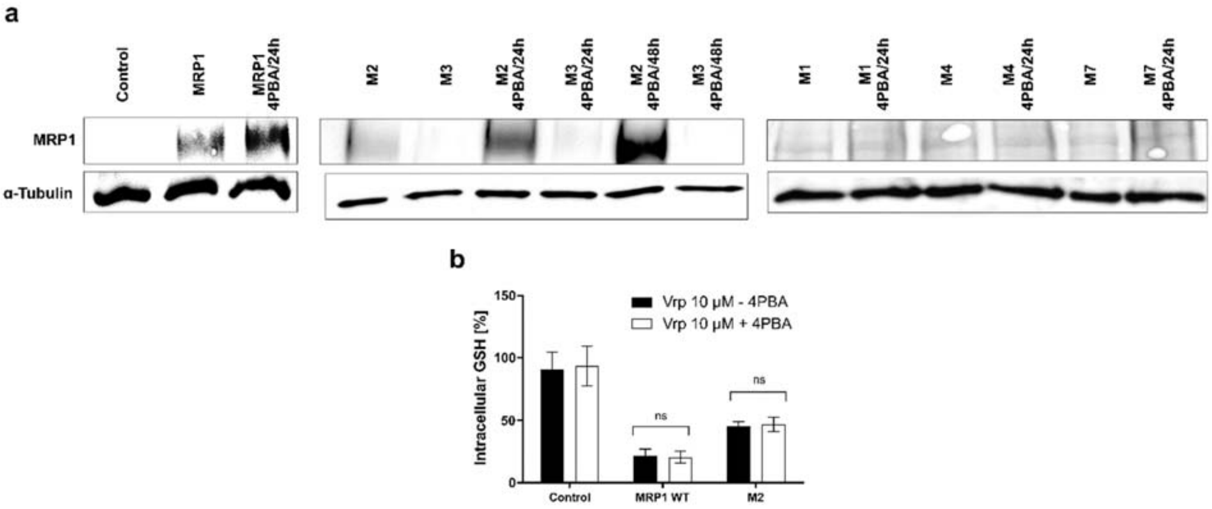

Supplementary Figure S3

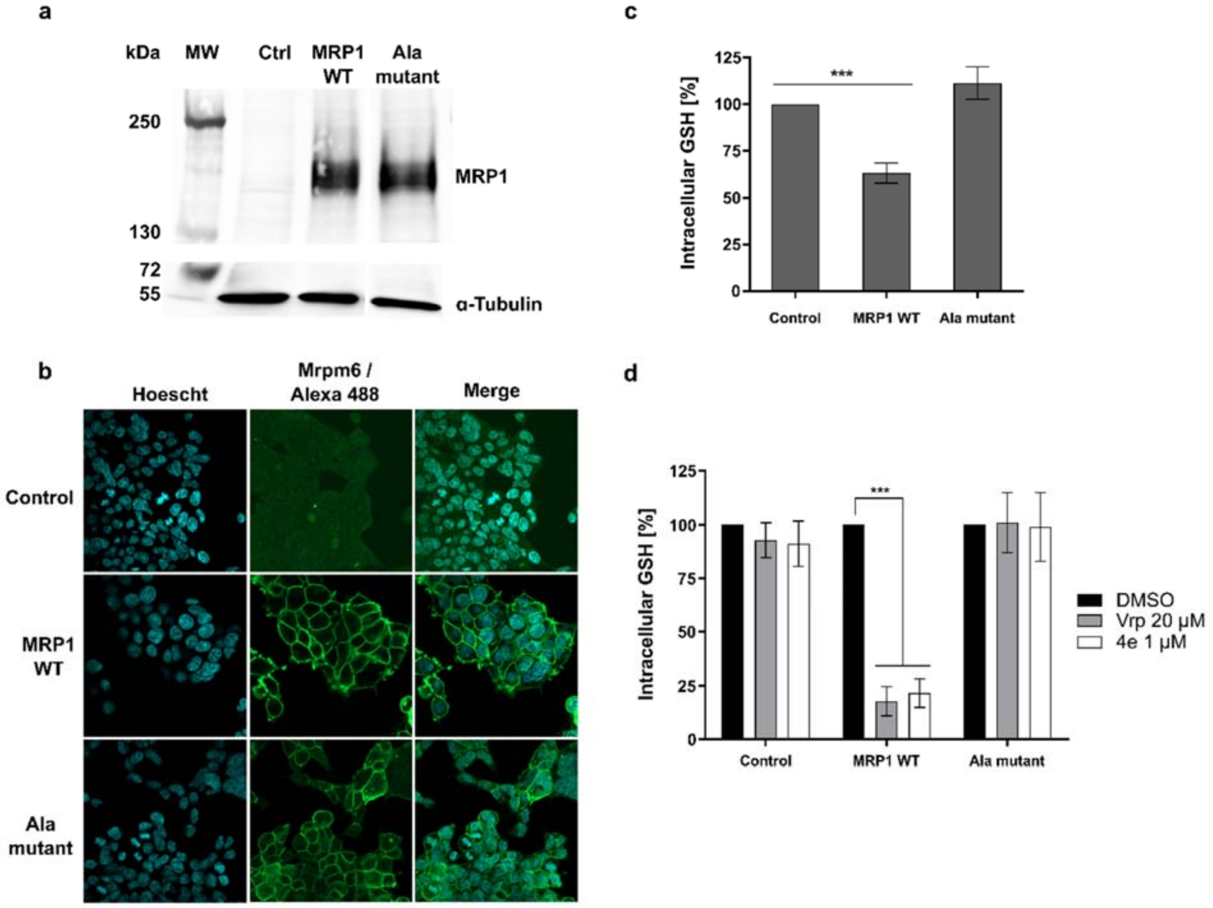

Supplementary Figure S4

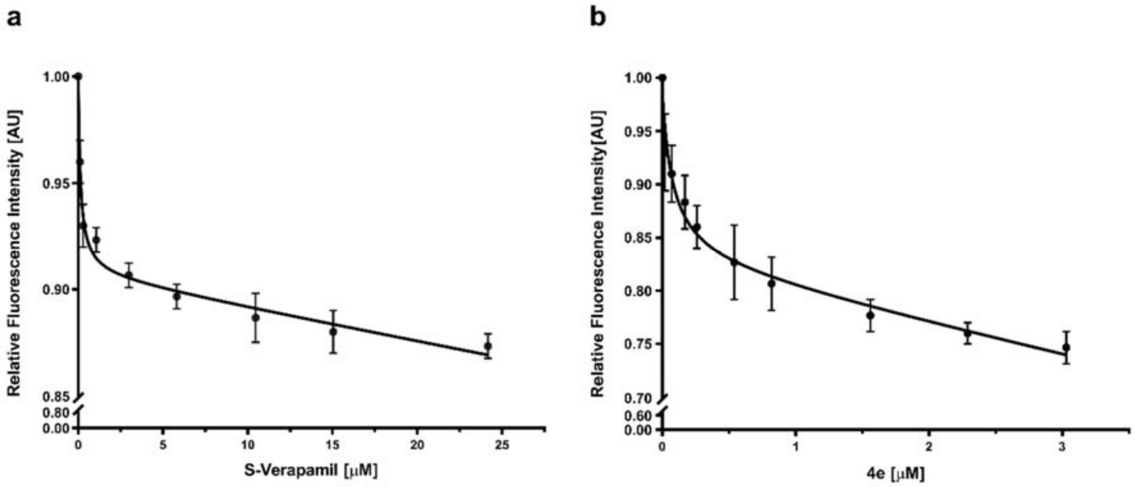

Supplementary Figure S5

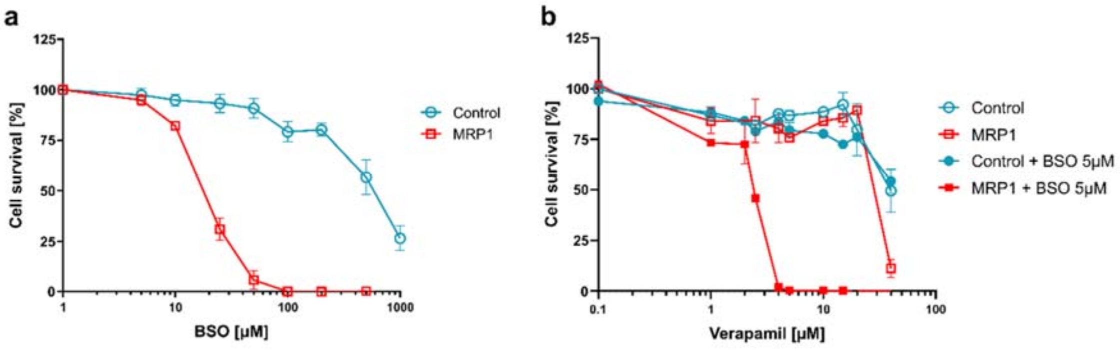

Supplementary Figure S6

a

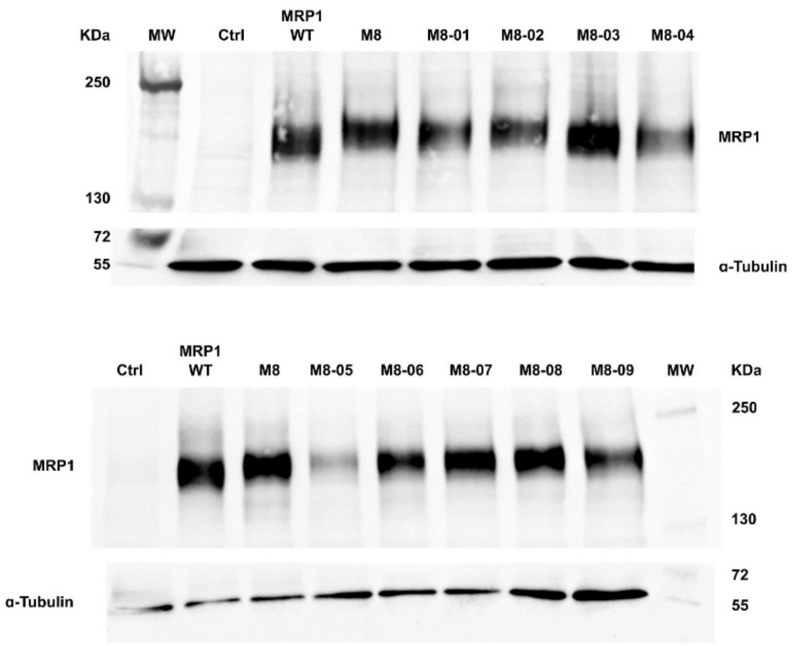

b

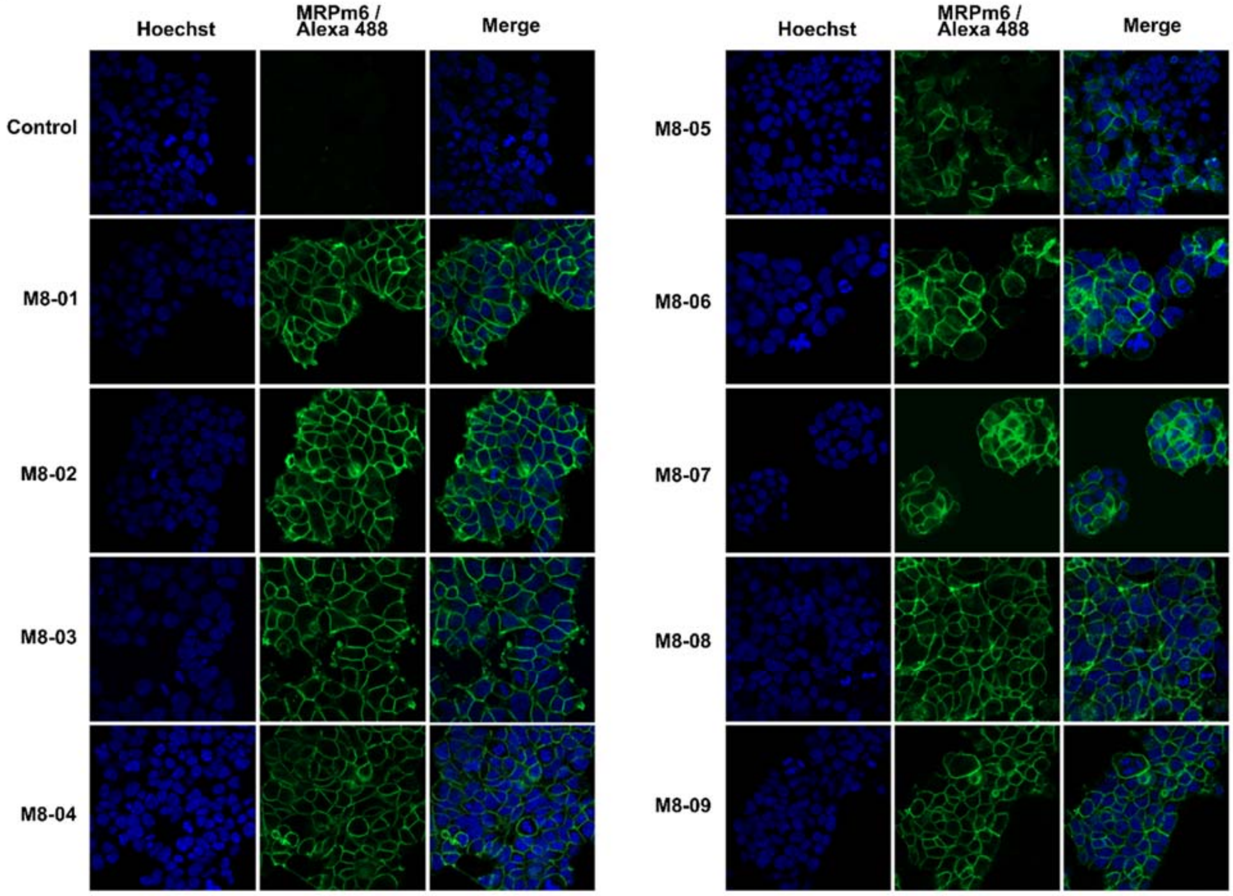
Hoechst
MRPm6 / Alexa 488
Merge
Control
M8-01
M8-02
M8-03
M8-04
Hoechst
MRPm6 / Alexa 488
Merge
M8-05
M8-06
M8-07
M8-08
M8-09

Supplementary Figure S7

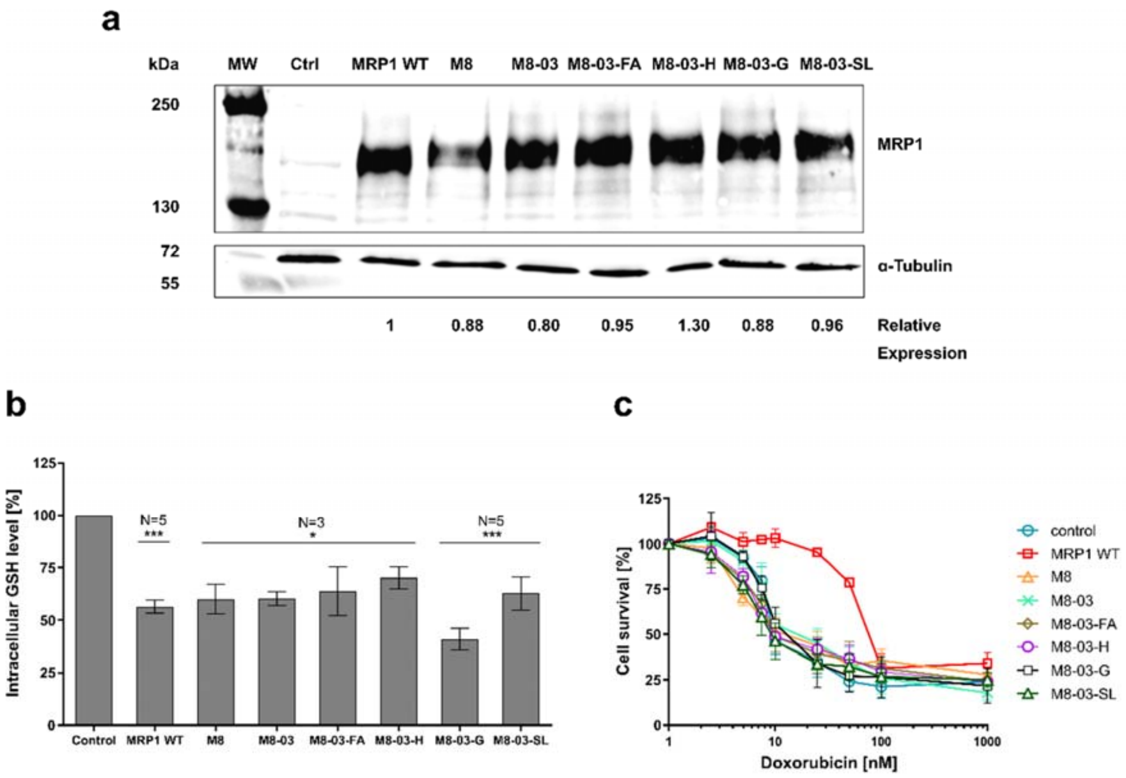

Original, unprocessed versions of the western-blots

Figure 1

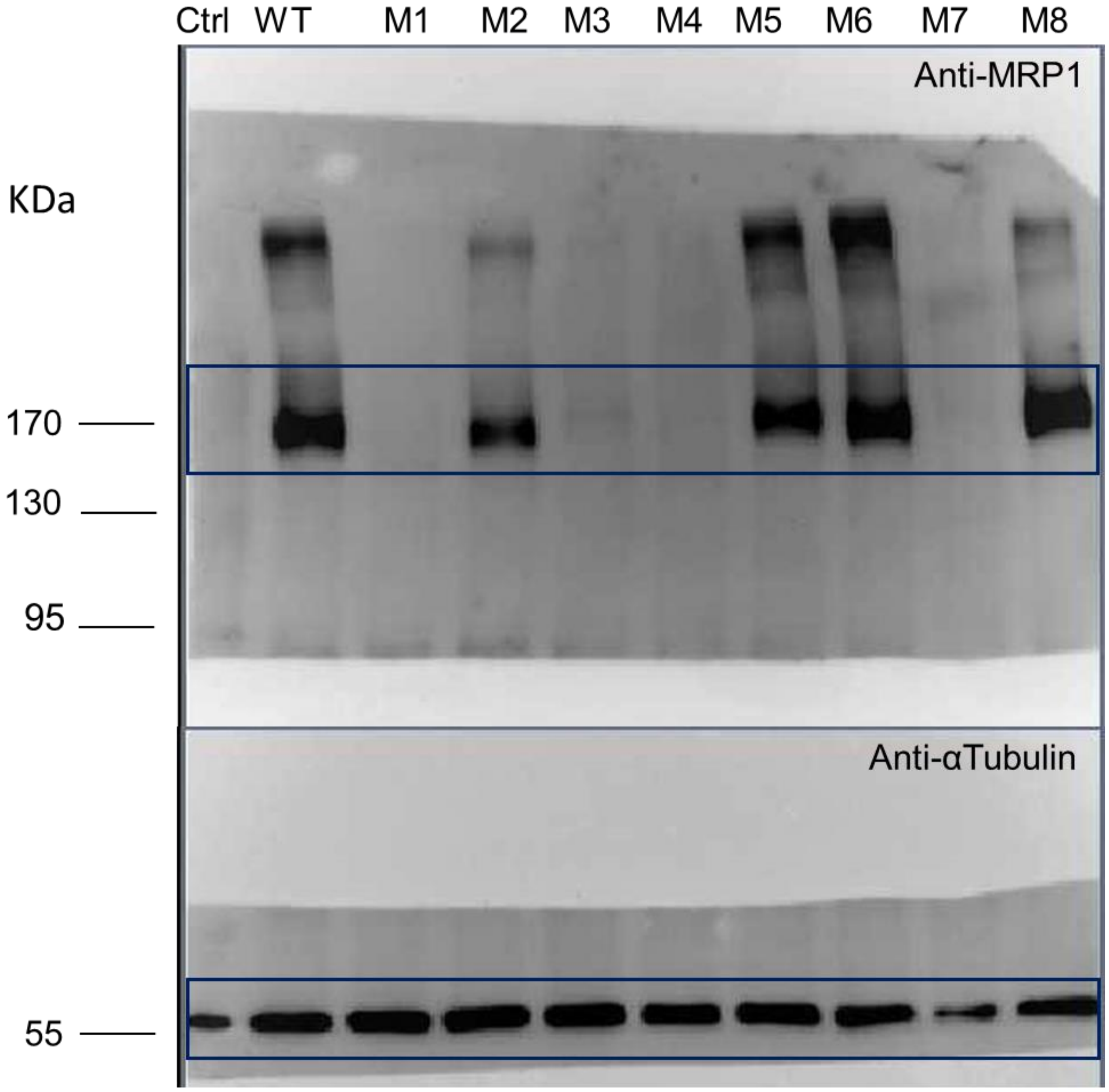

The nitrocellulose membrane was cut after the marker 95 kDa and the two parts were separately probed with either the anti-MRP1 monoclonal antibody MRPm6, or a polyclonal alpha-tubulin antibody as loading control. For the publication only the parts inside the blue squares were presented.

Supplementary Figure S1

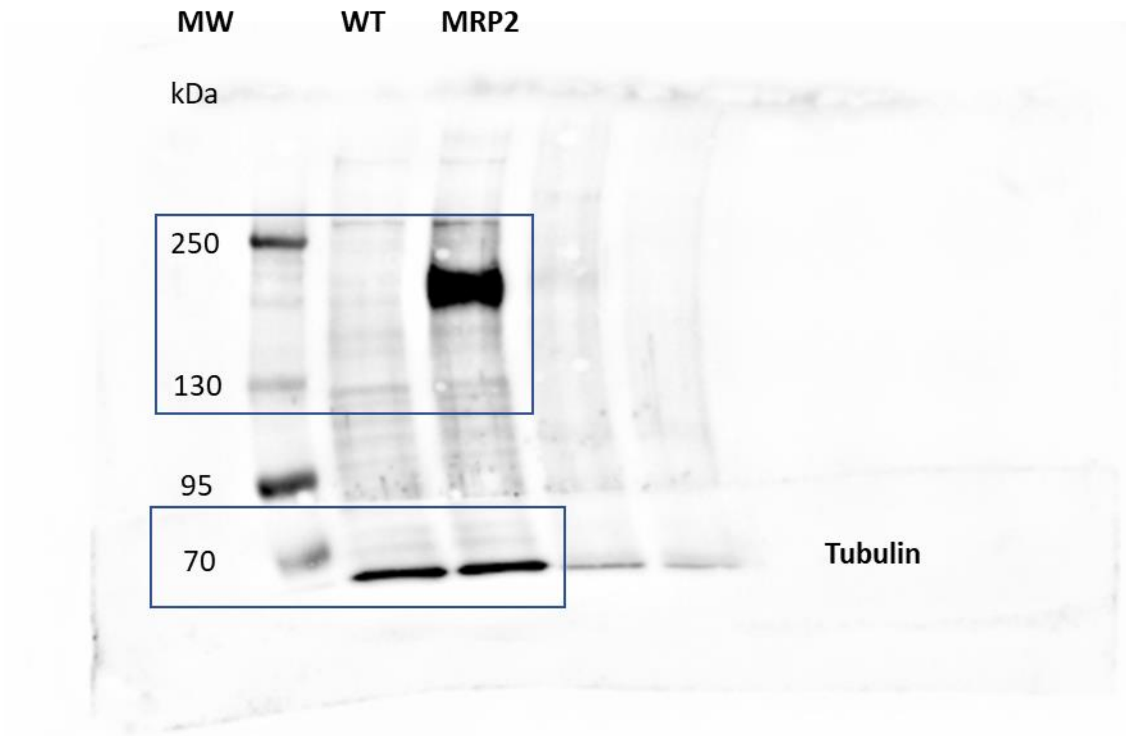

The nitrocellulose membrane was cut in between the marker 70 and 95 kDa and the two parts were separately probed with either the anti-MRP2 monoclonal antibody M2I-4, or a polyclonal alpha-tubulin antibody as loading control. For the publication only the parts inside the blue squares were presented.

Supplementary Figure S2a

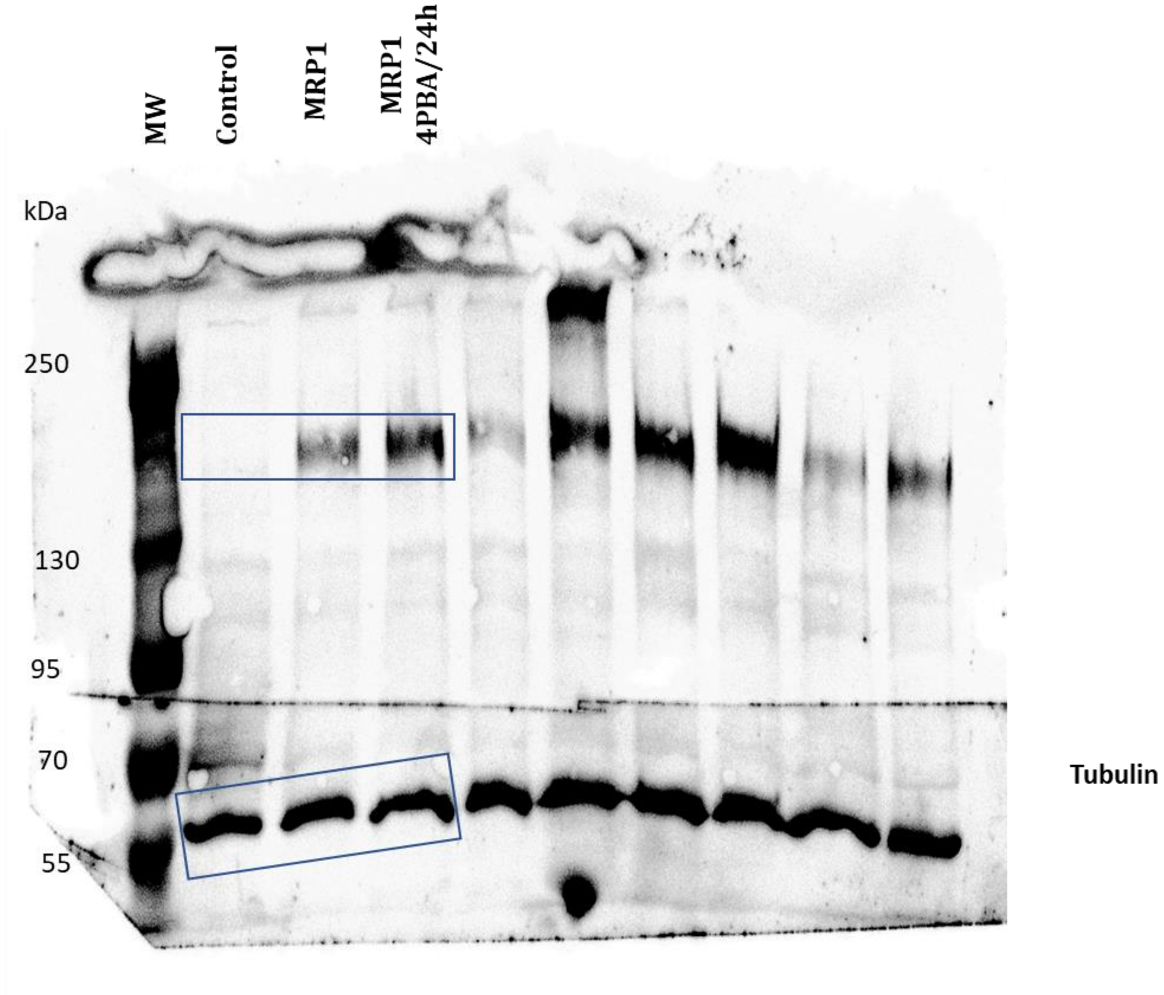

The nitrocellulose membrane was cut in between the marker 70 and 95 kDa and the two parts were separately probed with either the anti-MRP1 monoclonal antibody MRPm6, or a polyclonal alpha-tubulin antibody as loading control. For the publication only the parts inside the blue squares were presented in Figure S2a.

Supplementary Figure S2a

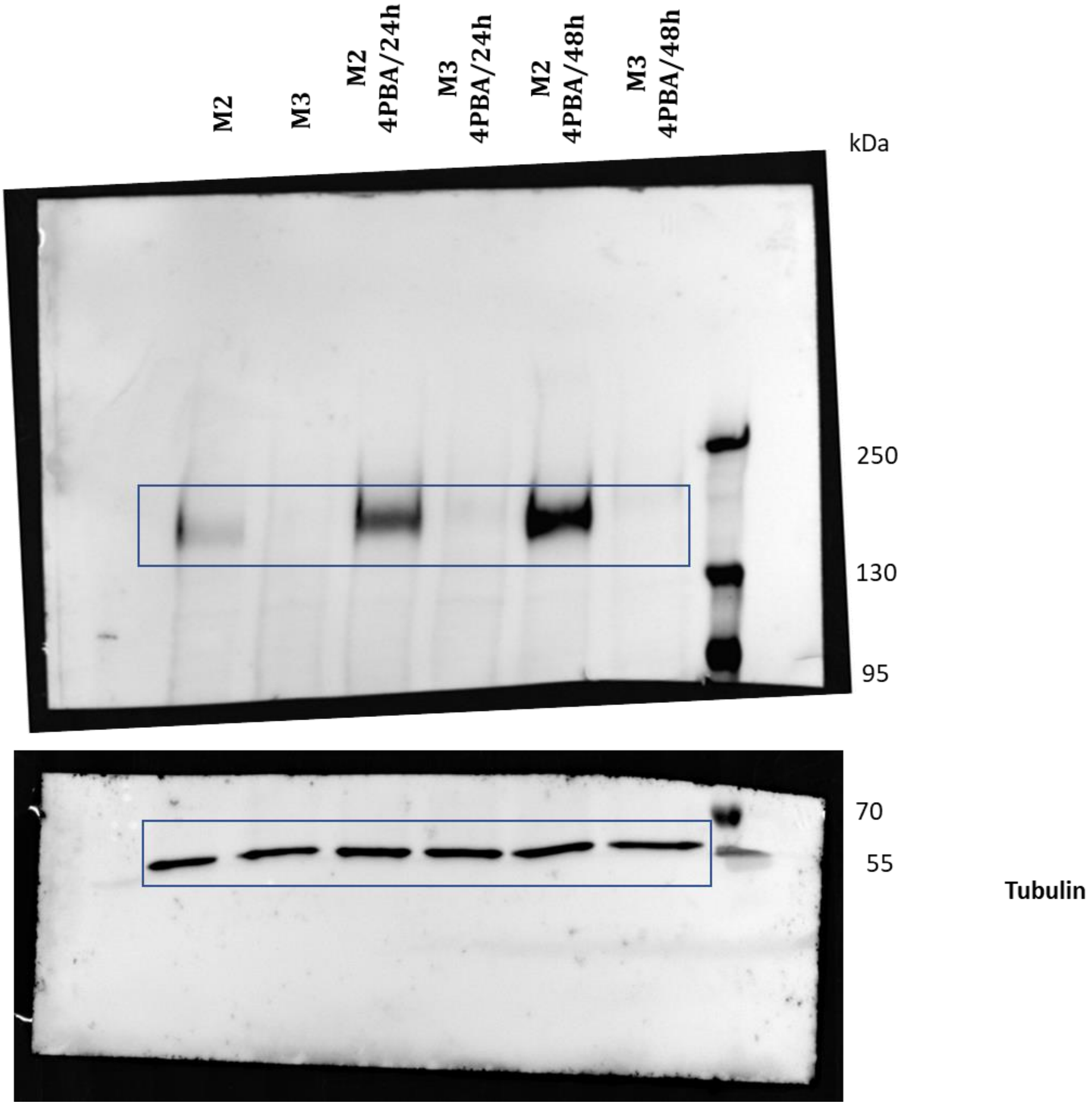

The nitrocellulose membrane was cut in between the marker 70 and 95 kDa and the two parts were separately probed with either the anti-MRP1 monoclonal antibody MRPm6, or a polyclonal alpha-tubulin antibody as loading control. For the publication only the parts inside the blue squares were presented.

Supplementary Figure S2a

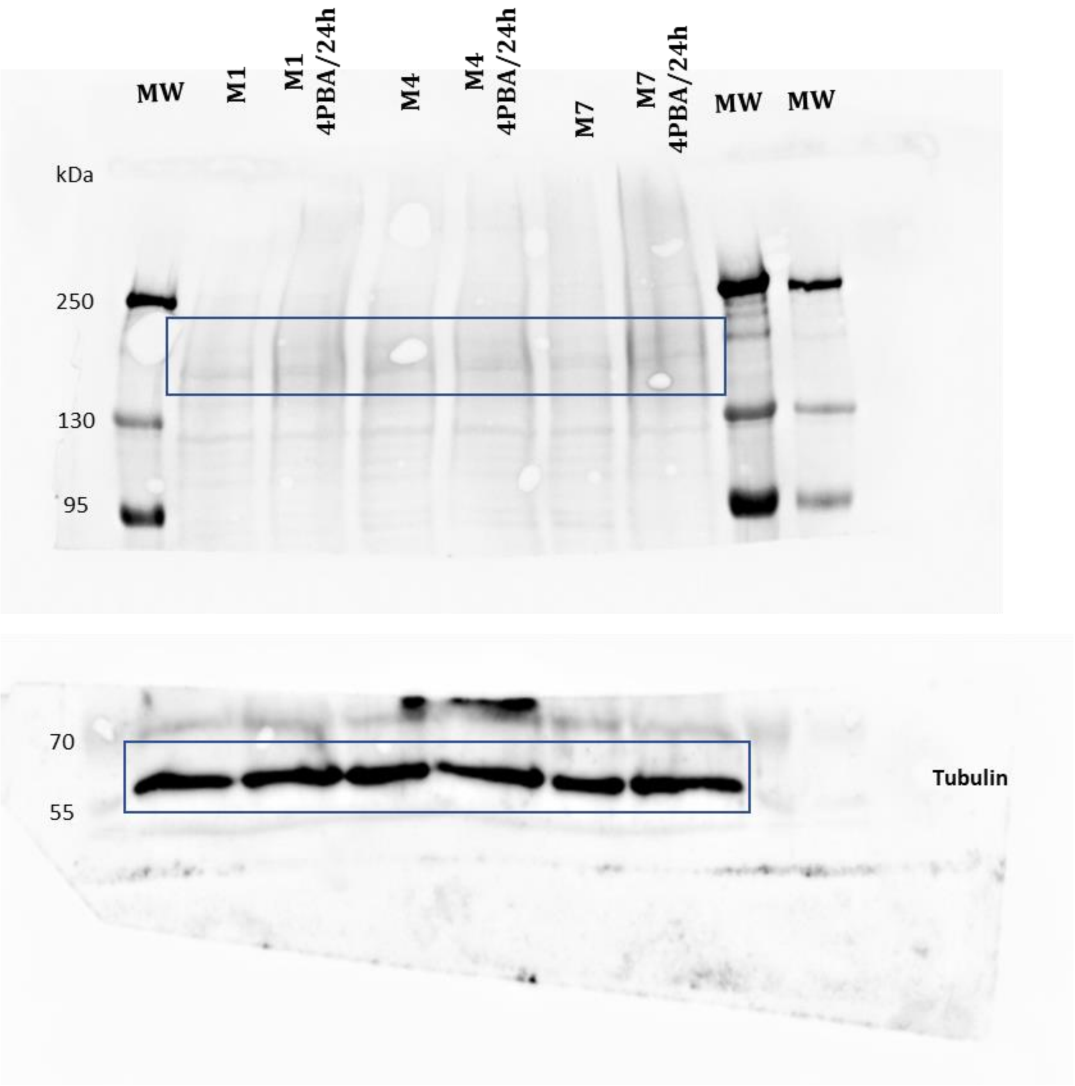

The nitrocellulose membrane was cut in between the marker 70 and 95 kDa and the two parts were separately probed with either the anti-MRP1 monoclonal antibody MRPm6, oa polyclonal alpha-tubulin antibody as loading control. For the publication only the parts inside the blue squares were presented.

Supplementary Figures S3a and S6a

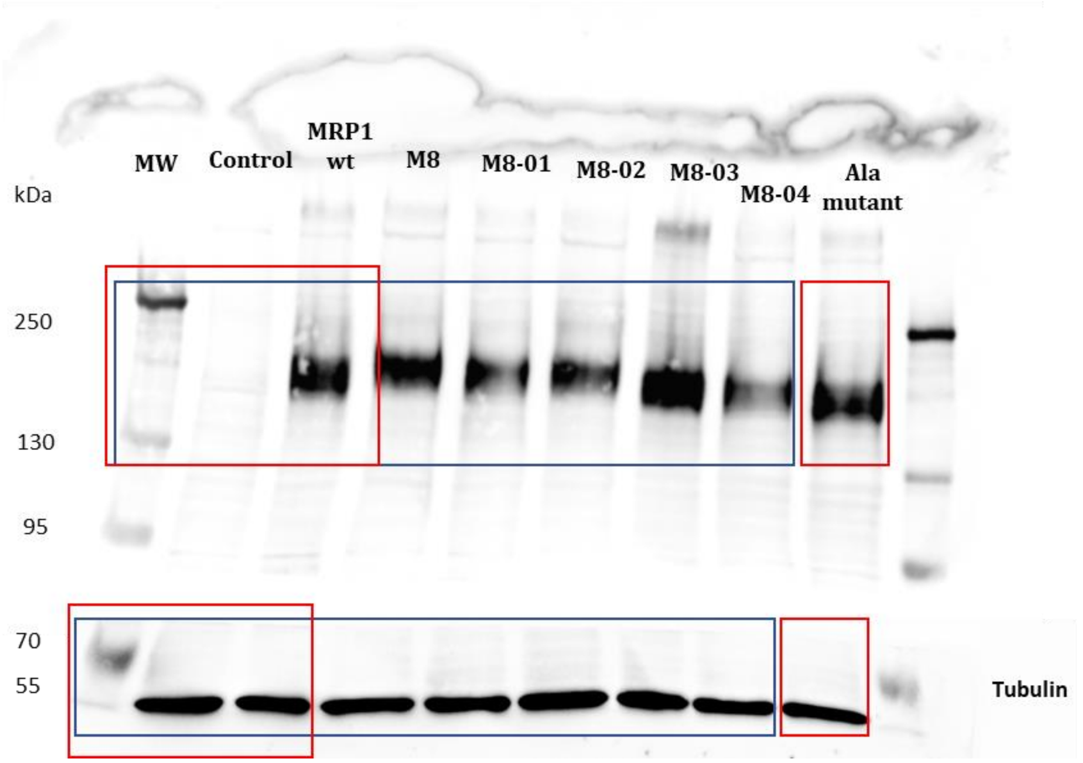

The nitrocellulose membrane was cut in between the marker 70 and 95 kDa and the two parts were separately probed with either the anti-MRP1 monoclonal antibody MRPm6, or a polyclonal alpha-tubulin antibody as loading control. For the publication only the parts inside the red squares were represented for figure S3a and inside the blue squares were presented for figure S6a.

Supplementary Figure S6a

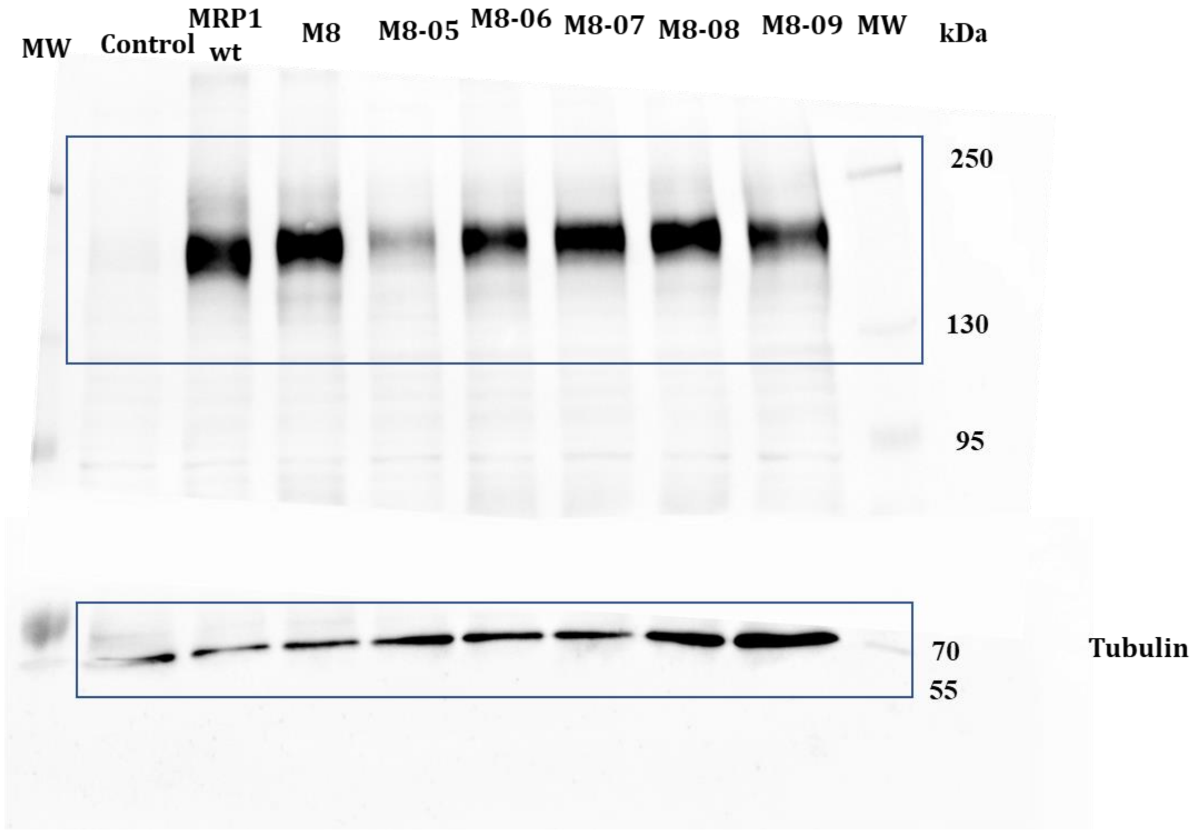

The nitrocellulose membrane was cut in between the marker 70 and 95 kDa and the two parts were separately probed with either the anti-MRP1 monoclonal antibody MRPm6, or a polyclonal alpha-tubulin antibody as loading control. For the publication only the parts inside the blue squares were presented.

Supplementary Figure S7

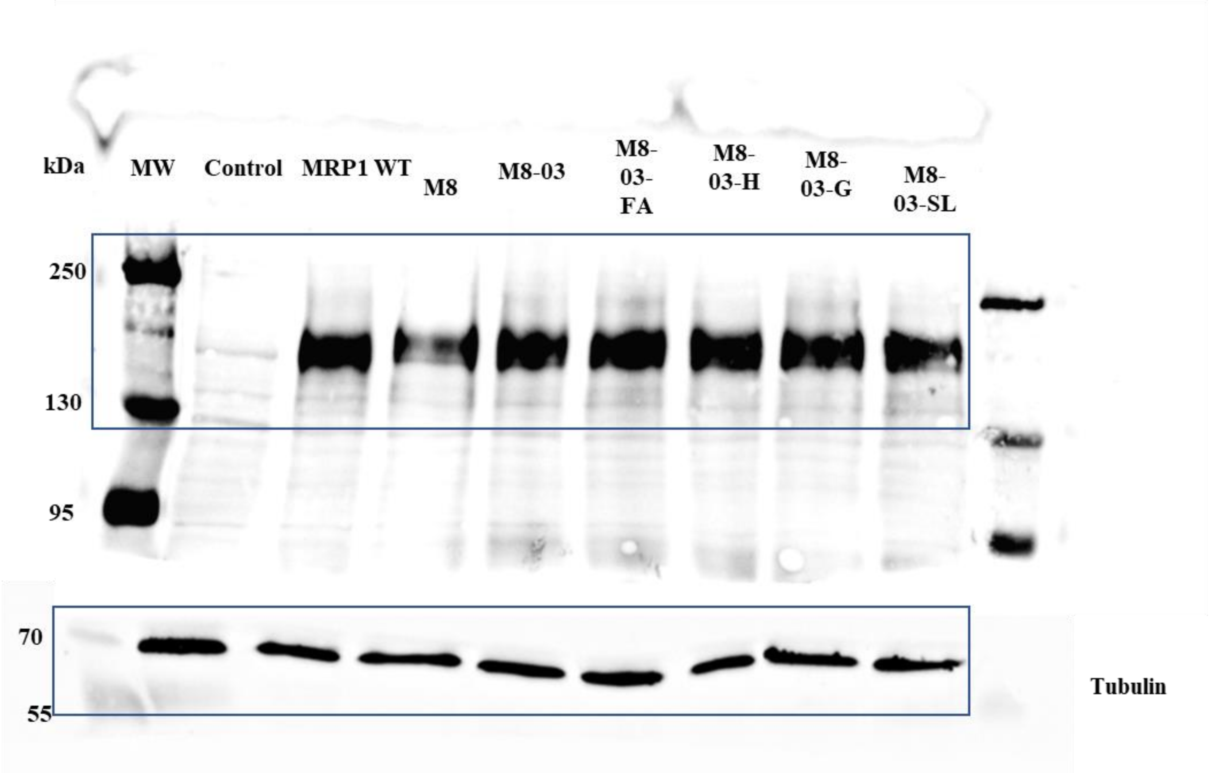

The nitrocellulose membrane was cut in between the marker 70 and 95 kDa and the two parts were separately probed with either the anti-MRP1 monoclonal antibody MRPm6, or a polyclonal alpha-tubulin antibody as loading control. For the publication only the parts inside the blue squares were presented.
